# Quantitative comparison between sub-millisecond time resolution single-molecule FRET measurements and 10-second molecular simulations of a biosensor protein

**DOI:** 10.1371/journal.pcbi.1008293

**Published:** 2020-11-05

**Authors:** Dylan Girodat, Avik K. Pati, Daniel S. Terry, Scott C. Blanchard, Karissa Y. Sanbonmatsu

**Affiliations:** 1 Theoretical Biology and Biophysics, Theoretical Division, Los Alamos National Laboratory, Los Alamos, New Mexico, United States of America; 2 Department of Structural Biology, St. Jude Children’s Research Hospital, Memphis, Tennessee, United States of America; 3 New Mexico Consortium, Los Alamos, New Mexico, United States of America; University of Maryland School of Pharmacy, UNITED STATES

## Abstract

Molecular Dynamics (MD) simulations seek to provide atomic-level insights into conformationally dynamic biological systems at experimentally relevant time resolutions, such as those afforded by single-molecule fluorescence measurements. However, limitations in the time scales of MD simulations and the time resolution of single-molecule measurements have challenged efforts to obtain overlapping temporal regimes required for close quantitative comparisons. Achieving such overlap has the potential to provide novel theories, hypotheses, and interpretations that can inform idealized experimental designs that maximize the detection of the desired reaction coordinate. Here, we report MD simulations at time scales overlapping with *in vitro* single-molecule Förster (fluorescence) resonance energy transfer (smFRET) measurements of the amino acid binding protein LIV-BP^SS^ at sub-millisecond resolution. Computationally efficient all-atom structure-based simulations, calibrated against explicit solvent simulations, were employed for sampling multiple cycles of LIV-BP^SS^ clamshell-like conformational changes on the time scale of seconds, examining the relationship between these events and those observed by smFRET. The MD simulations agree with the smFRET measurements and provide valuable information on local dynamics of fluorophores at their sites of attachment on LIV-BP^SS^ and the correlations between fluorophore motions and large-scale conformational changes between LIV-BP^SS^ domains. We further utilize the MD simulations to inform the interpretation of smFRET data, including Förster radius (R_0_) and fluorophore orientation factor (κ^2^) determinations. The approach we describe can be readily extended to distinct biochemical systems, allowing for the interpretation of any FRET system conjugated to protein or ribonucleoprotein complexes, including those with more conformational processes, as well as those implementing multi-color smFRET.

## Author summary

Förster (fluorescence) resonance energy transfer (FRET) has been used extensively by biophysicists as a molecular-scale ruler that yields fundamental structural and kinetic insights into transient processes including complex formation and conformational rearrangements required for biological function. FRET techniques require the identification of informative fluorophore labeling sites, spaced at defined distances to inform on a reaction coordinate of interest and consideration of noise sources that have the potential to obscure quantitative interpretations. Here, we describe an approach to leverage advancements in computationally efficient all-atom structure-based molecular dynamics simulations in which structural dynamics observed via FRET can be interpreted in full atomistic detail on commensurate time scales. We demonstrate the potential of this approach using a model FRET system, the amino acid binding protein LIV-BP^SS^ conjugated to self-healing organic fluorophores. LIV-BP^SS^ exhibits large scale, sub-millisecond clamshell-like conformational changes between open and closed conformations associated with ligand unbinding and binding, respectively. Our findings inform on the molecular basis of the dynamics observed by smFRET and on strategies to optimize fluorophore labeling sites, the manner of fluorophore attachment, and fluorophore composition.

## Introduction

Computational advancements have enabled the field of molecular dynamics (MD) simulations of biomolecular components to progress from sub-nanosecond to millisecond simulation time scales [1–3]. The combination of enhanced sampling algorithms and increased compute power has led to extended time scale simulations and rapid growth in the number of MD publications [1]. As MD simulations for studying conformational dynamics in biological systems venture into the realm of milliseconds and beyond, we are now at the precipice of MD simulation time scales that are temporally commensurate to an increasing number of *in vitro* experimental techniques. Congruence between MD and experimental time scales is also made increasingly possible as *in vitro* techniques advance towards collecting data at increasingly rapid time resolutions by leveraging improvements in laser, fluorophore, and detector performance [4–7]. Quantitative comparison of *in vitro* biochemical and spectroscopic data with *in silico* MD simulation on comparable time scales has the potential to provide rigorous structural and molecular interpretations of dynamic biomolecular events in atomic detail.

It is becoming standard practice in the field of MD simulations to perform explicit-solvent equilibrium simulations of small proteins in the microsecond range, while coarse-graining approaches have reached millisecond time scales [8,9]. Simulations have been used to predict chemical shifts and directly compare these results to nuclear magnetic resonance (NMR) experiments [10–23]. Simulations have also been used to recapitulate small angle X-ray scattering (SAXS) data [24–37]. Temporal information can be gleaned in NMR or SAXS by a diverse variety of means, including peak broadening in NMR, or by performing SAXS in time-resolved modes [38]. When data from these techniques are directly compared to MD simulations, the time scale of the simulations is often not considered critical, so long as sampling is sufficient to avoid equilibration artifacts [10–35]. Beyond these techniques, MD simulations can provide atomistic insights into structural dynamics for a multitude of experimental approaches [39–46]. Yet, close quantitative comparisons between *in vitro* experimental data and *in silico* simulations at commensurate time scales are relatively rare.

Single-molecule Förster Resonance Energy Transfer (smFRET), an experimental technique used to study the dynamics of a wide variety of biomolecules, from polymerases to ribosomes, to G-protein coupled receptors, could benefit greatly from quantitative MD simulation comparison [47–51]. Investigations involving MD interpretations of smFRET experiments have been a major focus for some time [52]. Recent pioneering studies utilized a 400 microsecond MD simulation in combination with machine learning and restraints derived smFRET experiments to recapitulate or interpret smFRET states and the transitions between them [53–56]. Multiple groups have also employed MD simulations to model FRET efficiencies derived from protein conformational ensembles [57–59]. Still others have used smFRET data to inform simulations of peptides [60]. To our knowledge, direct one-to-one comparisons of MD simulations with smFRET data of full-length proteins on congruent time scales have, however, yet to be performed.

To expand the predictive power of MD simulations for direct one-to-one comparisons with experiment, all-atom simulations must be performed on time scales that overlap with experimental observables (*i*.*e*., aggregate sampling of seconds). Explicit solvent simulations on the time scale of smFRET investigations are not trivial and can require hundreds of GPU core years. Explicit solvent simulations of small biological systems, such as a single protein, are also difficult to scale efficiently to supercomputers. For a 23,558 atom system, the Anton 2 supercomputer has proven capable of producing 85 μs/day [61]. To achieve explicit solvent simulations on Anton on time scales commensurate with smFRET, *i.e.* 10 seconds, MD calculations would, therefore, have to be performed continuously for several years.

To address this issue, we sought to leverage advances in the computationally economical, all-atom Gō-like structure-based models and to assess our findings with kinetic theory to compute rates [62]. More specifically, we employed a multibasin Gō-like structure-based simulation approach where multiple FRET states can be defined as native basins in the simulation, allowing for interconversion between states[63]. Simulations of this kind define contacts within a given state as a native basin, but allow for large-scale, spontaneous conformational changes [64,65]. For this technique, specific hydrogen bonds, electrostatic interactions, and rotamer angles are implicitly included in the native basin. By combining this approach with explicit-solvent equilibrium simulations, one can achieve comprehensive views of the dynamic ensemble of a biomolecule by explicitly resolving even more subtle interactions, such as explicit hydrogen bonds, electrostatics, or solvation effects [65,66].

Here, we take advantage of such methods to perform comparisons of MD simulations and smFRET data on commensurate time scales. This effort has been enabled by numerous advances in both fields of research, including the aforementioned breakthroughs in MD simulations and rapidly evolving smFRET instrumentation, self-healing organic fluorophores, and the identification of the amino acid binding protein leucine-isoleucine-valine binding protein (LIV-BP) as a robust model system [5,67–69]. This biosensor, with the inclusion of mutations to remove native cysteines (LIV-BP^SS^), rapidly binds leucine, isoleucine, and valine amino acids with a k_on_ of 30 μM^-1^ s^-1^ for Leu [68]. We use this system to develop a pipeline for recapitulating smFRET data *in silico* for a direct structural interpretation of the underlying conformational processes associated with amino acid binding and unbinding. We then employ kinetic theory to determine rates of transitions between FRET states to enable direct comparisons between our *in silico* and *in vitro* results [62]. Using all-atom structure-based simulations on comparable time scales to *in vitro* experiments, together with explicit solvent simulations on shorter time scales, we examine the key variables and determinants of smFRET data, including quantitative descriptions of Förster radius (R_0_) and fluorophore orientation factor (κ^2^) parameters that inform on the FRET-distance relationship. Importantly, this pipeline is not restricted to LIV-BP^SS^. It can be reconstituted for any smFRET assay to gain all-atom structural perspectives on experimental data.

## Results

### Single-molecule FRET experiments and molecular simulations resolve LIV-BP^SS^ conformational changes on commensurate time scales

**Fig 1:**
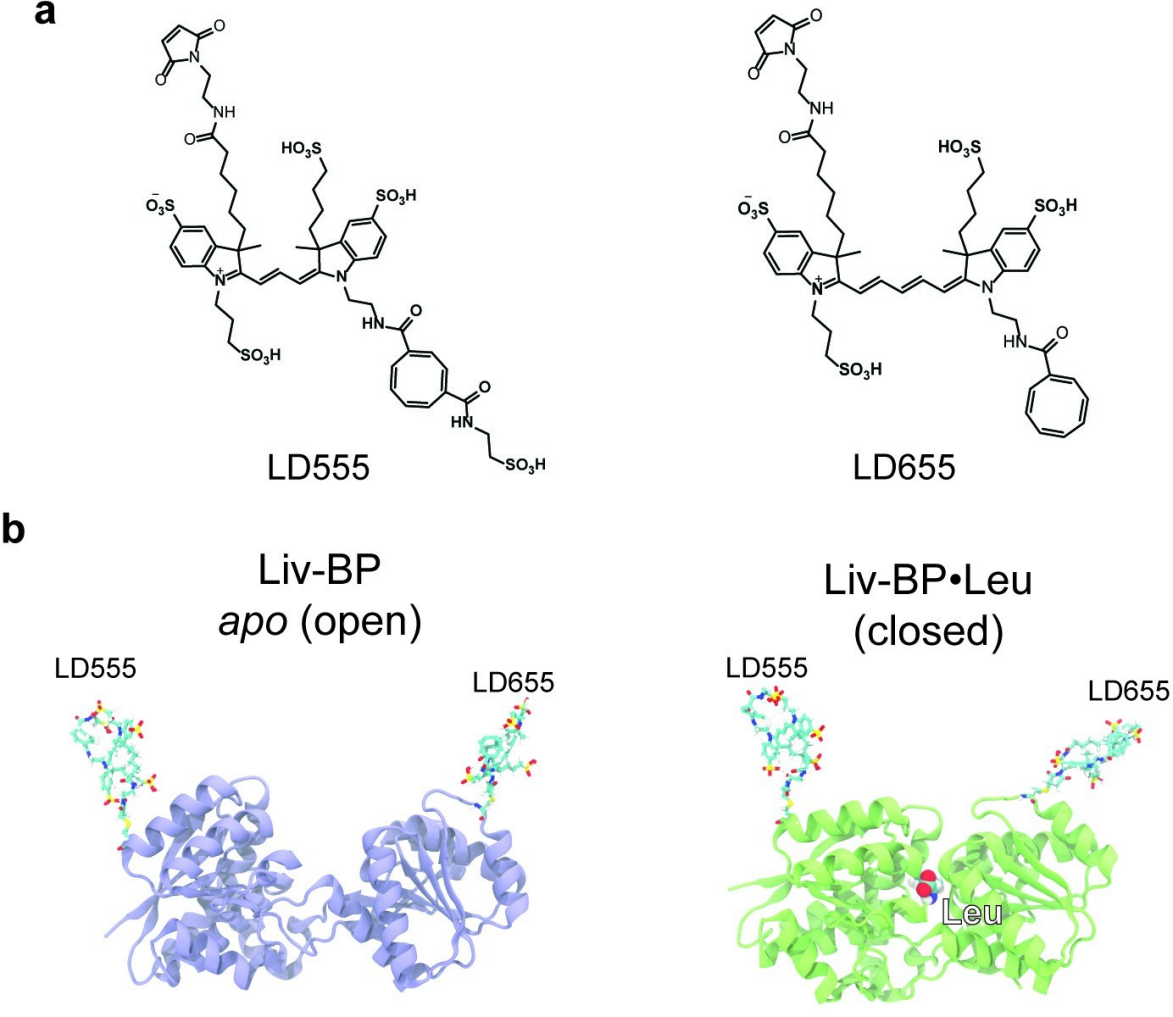
Models of Liv-BP^SS^ conjugated to self-healing fluorophores. (a) Molecular structures of the LD555 and LD655 fluorophores. (b) Conformation of Liv-BP^SS^ in the open (*apo*) conformation–blue, and closed (Leu-bound) conformation–green. For multibasin Gaussian potential simulations the open (*apo*) conformation and the closed (Leu-bound) conformations were set as native states. The models were also used as initial structures for explicit solvent simulations.

**Fig 2:**
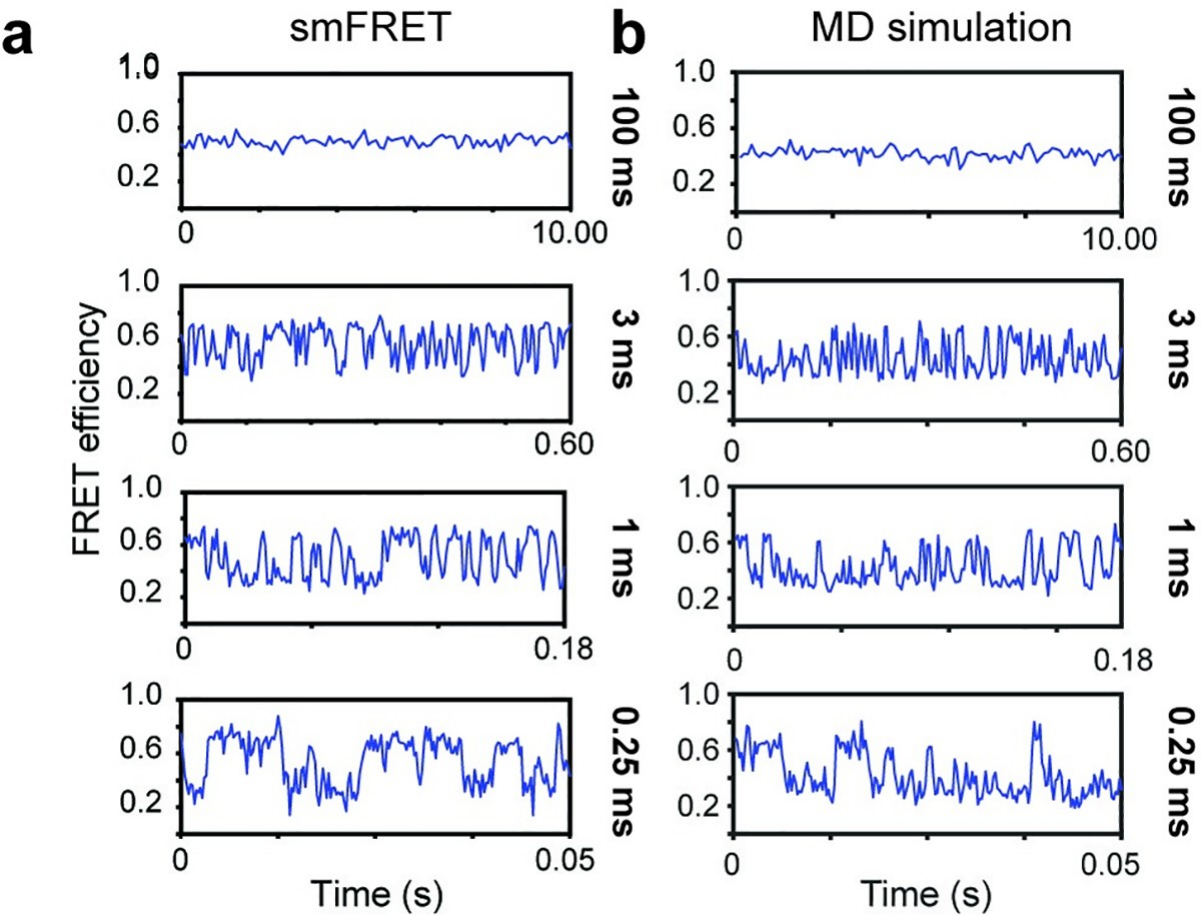
Recapitulation of experimental smFRET data from 200 simulations at 500 million-timesteps (total of 100 billion-time steps). (a) Representative experimental smFRET traces of the LIV-BP^SS^ system at 0.25, 1, 3, and 100 ms exposure times [70]. (b) Representative FRET trajectories from MD simulations (including dye-linker interactions, see below) at the same exposure times as smFRET experiments. Effective exposure times for the MD simulations were controlled by averaging together the FRET efficiency values of consecutive time steps; for example, each data point in the 100 ms exposure is an average of 4x10^9^ time steps. For all MD simulations and smFRET experiments, fluorophores were conjugated to position 67 and 181 of LIV-BP^SS^.

The LIV-BP model system employed here for smFRET experiments and MD simulations corresponds to the LIV-BP^SS^ protein described previously [68]. Previous experimental investigations using a fluorescently labeled LIV-BP^SS^ protein have shown that ligand binding rates approach the diffusion limit (ca. ~10^8^ M^-1^ s^-1^) [68]. In the present study, analogous to prior investigations [68,70], LIV-BP^SS^ was labelled at positions 67 and 181 by mutating native residues to Cys and performing maleimide chemistry with the self-healing fluorophores LD555 and LD655 (Fig 1A). In the MD simulations, LD555 and LD655 fluorophores were explicitly modeled at these same positions. As previously described [68], the sites of labeling in LIV-BP^SS^ were selected based on X-ray crystal structures of ligand-bound (closed conformation) and ligand-free (open conformation) states (PDB ID: 1Z15, 1Z16, 1Z17, and 1Z18) (Fig 1B) [71] to report on the conformational changes upon amino acid binding and dissociation, respectively. This conformational change can be described as a “clamshell” or “fly-trap” motion, where the two domains of LIV-BP^SS^ approach each other in the ligand-bound state (closed conformation) (Fig 1B). Capturing the conformational dynamics between the closed and open LIV-BP^SS^ conformations using smFRET require camera exposure times below 5 ms (Fig 2A) [68,70]. Notably, typical explicit solvent MD simulations have time steps of 1–4 fs. By contrast, reduced-description MD simulations can exhibit up to nanosecond individual time steps. Thus, any smFRET time resolution can, in principle, be matched with MD simulations. However, to simulate the large number of conformational transitions observed in smFRET experiments, time scales on the order of hundreds of milliseconds to seconds are required. Such an undertaking is not trivial to achieve.

For comparison to MD simulations, we examined the conformational changes of LIV-BP^SS^ in smFRET experiments at varied camera frame rates (100 ms to 0.25 ms) (Fig 2A) [70]. To ensure a robust comparison between experimental FRET efficiencies and those derived from simulations, we corrected the experimental smFRET data for spectral crosstalk, relative detection efficiencies and quantum yields (Q_D_), and acceptor direct excitation (see Methods). Notably, in the presence of the leucine (Leu) ligand at the K_D_ of ligand binding (~4.5 μM) in the 100 ms exposure regime, only one FRET state is evidenced that exhibits ~0.52 FRET efficiency (see Methods). This finding reflects time-averaging effects related to the rapid association and dissociation rates of leucine to LIV-BP^SS^(~30 μM^-1^ s^-1^ and ~212 s^-1^, respectively) [68,70]. Consistent with this interpretation, at exposure times of 3 ms and below, two distinct FRET states exhibiting ~0.65 and ~0.35 FRET efficiency, corresponding to open and closed LIV-BP^SS^ conformations, respectively, are resolved (Fig 2A).

To perform MD simulations of LIV-BP^SS^ congruent with these experimental studies, site-specifically labelled LIV-BP^SS^ proteins, as described above, were used in dual-basin all-atom structure-based simulations to describe conformational changes between open and closed conformations (Fig 2B). In addition, explicit solvent simulations of *apo* and Leu-bound states were performed for an in-depth investigation of the dynamics of LIV-BP^SS^ near the open and closed basin minima. Structure-based simulations in the presence of Leu were used to observe transitions between the open and closed conformations. However, due to technical considerations related to the structure-based potential, Leu binding and unbinding events were not explicitly simulated (S1 Text). Because the ligand itself contributes relatively few native contacts to the bound (closed) state, this exclusion has little effect on the number and time scale of simulated transitions.

An advantage of structure-based potentials is that they can be relatively easily calibrated to experimental systems. Here, the structure-based potentials were calibrated to the opening and closing events observed at the K_D_ of Leu binding (~4.5 μM) to LIV-BP^SS^ in smFRET experiments. This approach enabled us to simplify the simulations, while recapitulating LIV-BP^SS^ opening and closing dynamics. In so doing, we were able to clearly define a double Gaussian potential for the LIV-BP^SS^ protein, where each basin is defined by the native contacts present in both the open and closed conformations (S1 Fig). In this approach, both conformations are set as native basins, allowing for the protein to transition between each conformation through barrier crossing events. Previous implementations of Gaussian basins involved studying kinetic processes such as protein folding and conformational changes [72,73]. By contrast, our simulations using the double Gaussian potential are expected to provide information regarding the conformational changes of LIV-BP^SS^. This implementation also enables analyses of LD555 and LD655 fluorophore dynamics, tumbling behaviors, and the simulation of FRET efficiencies based on the measured inter-fluorophore distances according to Förster theory.

Proper conversion of FRET efficiency values to fluorophore distances, or *vice versa*, requires accurate determination of the fluorophore positions in the system in three-dimensional space. To characterize the fluorophore distance correlation between MD simulations and smFRET experiments, we measured the distance in the MD simulations between the centers of mass of the explicit LD555 and LD655 chromophores (R_dye_) attached to positions 67 and 181 of the protein, respectively (S2 Fig). As expected, the fluorophore distances were observed to fluctuate, where the dye center of masses exhibited mean inter-fluorophore distance values of 69 ± 5 Å and 56 ± 8 Å for the open and closed conformations, respectively.

Precisely ascertaining the time interval of each time step of any simulation based on a reduced description potential is non-trivial. In the case of structure-based potentials, the time intervals are dependent on the size of the system, the number of particles, the contact weights, and the harmonic potentials employed. However, through direct comparison with *in vitro* experiments or explicit solvent simulations, estimates of the time scales for structure-based simulations can be achieved [65,74]. To estimate the time scales of the simulated LIV-BP^SS^ conformational changes, we directly compared the simulated dwell times of the closed conformation evidenced in our structure-based simulations to the corresponding dwell times observed in high-FRET state by smFRET.

**Fig 3:**
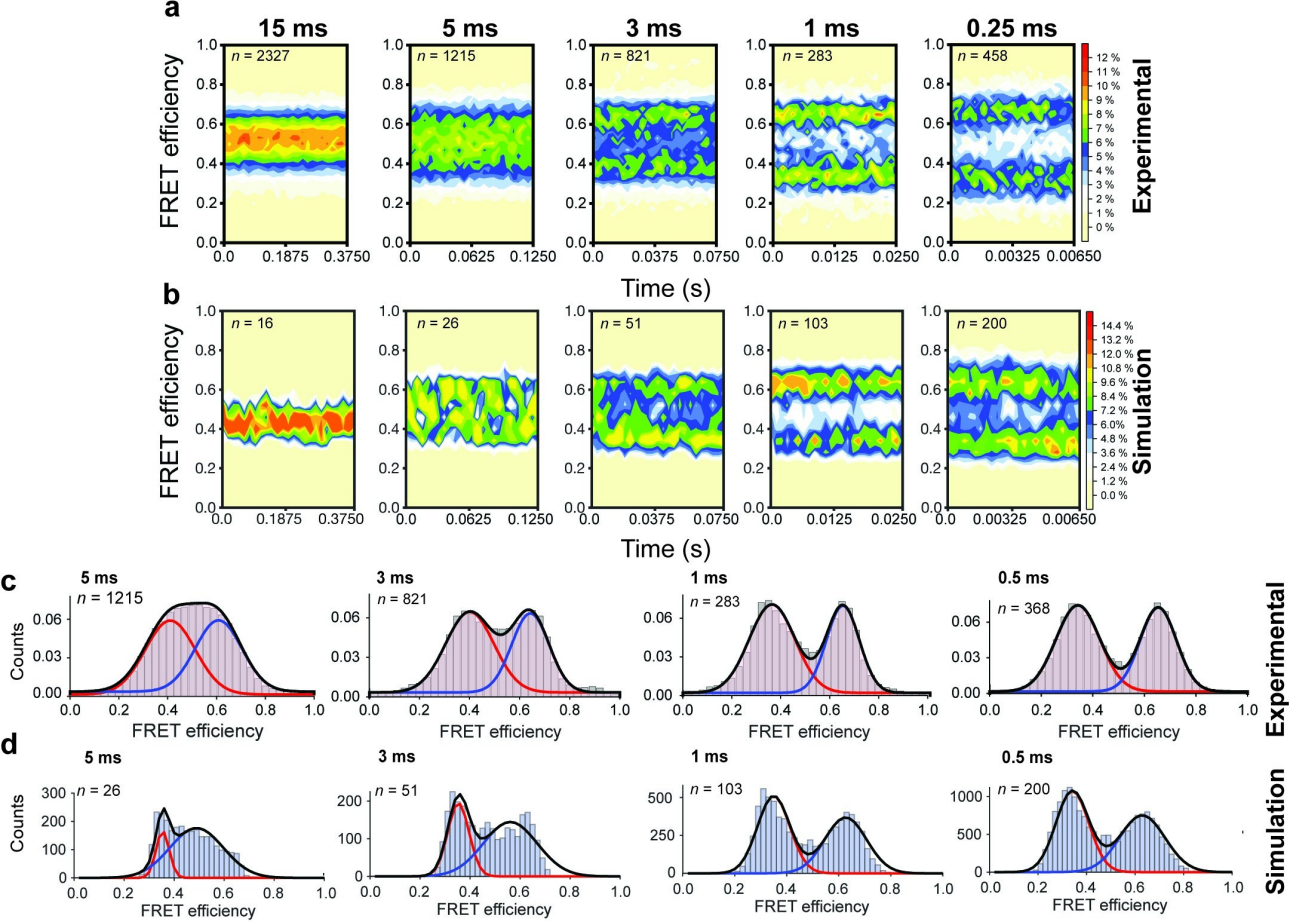
Population level correlation of smFRET and MD simulation FRET distributions. (a) smFRET traces were summed into FRET-time contour plots to show the distribution of FRET populations at 15, 5, 3, 1, and 0.25 ms camera exposure times [70]. (b) Contour plots of MD simulations at the equivalent exposure times (to achieve required sampling for 15ms exposure, 7 simulation trajectories were combined into one trajectory). (c) Histograms of the smFRET experiments (bars) fitted to a two gaussian functions (lines) to identify distinct states with data obtained using TIRF imaging of immobilized particles [70]. (d) Histograms of MD simulations at the equivalent exposure times as smFRET experiments. At 5 and 3 ms sampling, a 3 Gaussian fit was used as an intermediate population between the two FRET states is observed.

At the K_D_ of Leu binding (4.5 μM), LIV-BP^SS^ spends approximately 50% of its time in both open and closed conformations (Fig 3A–3C) [68,70]. The experimentally estimated opening rate under these conditions (~212 s^-1^) corresponds to Leu unbinding [68]. This measurement indicates that the average duration of the Leu-bound state is approximately 4.7 ms. By definition of the K_D_, the average lifetime of the open conformation must, therefore, also be ~4.7 ms. These data are globally consistent with the estimated K_D_ (~4.5 μM) and a near diffusion-limited ligand binding rate (~10^8^ M^-1^s^-1^).

The average dwell time of the closed conformation in the MD simulations was 1.9 ± 0.19 x 10^7^ time steps of simulation time (Fig 2B). This dwell time was determined by measuring the number of time steps LIV-BP^SS^ stays in the closed conformation (R_dye_ < 60 Å), where 0.19 x 10^7^ corresponds to the standard deviation of this value. Considering the dwell times evidenced in smFRET experiments, we correspondingly infer that each time step reflects approximately 0.25 ns. These estimates, which relate the time steps in structure-based models to physiological time scales, are similar in nature to those reported by Yang, *et al* for structure-based simulations, which estimated each simulated time step to be 0.05–1 ns [74].

A key determinant of the FRET-distance relationship is the fluorophore orientation factor (κ^2^), which relates to the relative orientation of the transition dipole moments of each fluorophore. The transition dipole moments of individual fluorophores are often assumed to be randomized by rotational diffusion. In such cases, the average fluorophore orientation is approximately isotropic, yielding a κ^2^ value of 2/3. Accurate κ^2^ values are required to accurately calculate the Förster distance (R_0_).

Fluorophore conjugation to a biomolecule can, however, influence its tumbling behaviors due to steric restrictions and hydrophobic and/or electrostatic effects. Correspondingly, a fluorophore’s position may be non-random. To ascertain if the fluorophores conjugated to LIV-BP^SS^ are randomly orientated, we first determined the transition dipole moment of the fluorophores with quantum mechanical (QM) calculations (S3 Fig). Using these dipole moments in conjunction with our structure-based simulations, we determined that the self-healing LD555 and LD655 fluorophores conjugated to LIV-BP^SS^ have κ^2^ = 0.58 ± 0.22, close to the theoretical 2/3 value (S1 Table). From explicit solvent simulations of open and closed LIV-BP^SS^ conformations performed in triplicate, we find κ^2^ values of 0.45 ± 0.19 and 0.41 ± 0.10, respectively. The lower estimation of κ^2^ from explicit solvent simulations is likely due to the limited sampling of fluorophore positions during the 1 μs simulation. The deviations of κ^2^, calculated in structure-based simulations (0.58 ± 0.22) from the ideal value (0.66) is expected to shorten R_0_ by 1 Å. More skewed fluorophore orientations would have greater effects. Such findings are consistent with the notion that the tumbling behaviors of fluorophores at their sites of attachment can influence estimations of the FRET-distance relationship, albeit modestly in the case of LIV-BP^SS^. Using the calculated κ^2^ value (0.58) based on structure-based simulations, and the experimentally determined LD555 quantum yield attached to LIV-BP^SS^ (0.48; see Methods), we estimated the R_0_ of the LD555/LD655 pair to be approximately 62.0 Å. This finding is in good agreement with the inter-fluorophore distance (R_dye_) changes estimated from our structure-based MD simulations of 69 ± 5 Å (open) and 56 ± 8 Å (closed) and the experimentally derived FRET efficiencies (~0.65 and 0.35 FRET, respectively), which are above and below 50% transfer efficiency for the LD555/LD655 pair (~62.0 Å) (discussed below).

### Correlating smFRET efficiencies with computed FRET efficiencies from MD simulations

Using the estimated duration of the simulated time steps (0.25 ns) and the estimated R_0_ (62.0 Å), we generated simulated FRET efficiency traces from the all-atom structure-based MD simulations (S4 Fig). As expected from experiment, simulated smFRET efficiencies at 1 ms exposure time (averaging 4 x 10^6^ time steps to reach 1 ms exposure) exhibited step-like transitions between high- and low-FRET states. However, the averaged FRET values were higher than those expected from smFRET experiments (~0.65 and ~0.35 for the experimentally observed open and closed conformations, respectively) (Figs 2A and 3A). We note in this context that the open and closed LIV-BP^SS^ conformations in our all-atom structure-based MD simulations, exhibit estimated FRET efficiencies of ~0.84 and ~0.47, respectively, roughly 0.1–0.2 higher FRET efficiency than those observed experimentally (S4 and S5 Figs.). In considering the origins of this difference, we noted that the structure-based simulations did not include the potential intramolecular contacts for the fluorophores observed in our explicit solvent simulations. As a result, LD555 and LD655 were highly dynamic and extended outward from the protein surface throughout the structure-based simulations, similar to antennae from an ant.

To examine the hypothesis that such a scenario may be unrealistic—for instance, by discounting fluorophore-linker or fluorophore protein interactions—we added contacts between the fluorophore and its linker (dye-linker interactions), which we discerned from our three, 1 μs explicit solvent simulations of LIV-BP^SS^ in apo and Leu-bound states. Here, fluorophore atoms that come within 3 Å of a linker atom were considered and added as a native contact to the structure-based simulations. This analysis led to the addition of 111 native contacts in the structure-based model for both fluorophores combined (S5 Fig). In the explicit solvent simulations, fluorophore atoms approach the protein as well. Nevertheless, these interactions were an order of magnitude fewer (39) in number and they were observed to be relatively transient in nature. We therefore did not consider them likely to significantly alter the observed fluorophore dynamics to the same extent as the intramolecular fluorophore-linker contacts. For example, the longest enduring interaction (distance shorter than 3 Å) between LD555 and the protein lasted for < 500 ns, and was only formed in one of the 6 explicit solvent simulations. By contrast, dye-linker interactions were observed in all explicit solvent simulations, most of which maintained for > 500 ns in both the open and closed conformations. Correspondingly, interactions between the fluorophores and protein were not considered for the structure-based simulations. The inclusion of the fluorophore-linker interactions, used to yield more accurate FRET values do not significantly impact the κ^2^ values (S1 Table), yet were introduced to provide a more precise fluorophore-fluorophore distances.

To examine the impacts of these contacts on the average fluorophore positions in our structure-based simulations, we scaled their strength from 0.1 to 1.0. Using this procedure, we empirically determined that a scaling of 0.5 for dye-linker contacts closely recapitulated the FRET values of the experimental traces (Fig 2A and 2B). At 100 ms exposure time for both the smFRET and MD simulations, only a single FRET population at ~0.5 FRET efficiency was observed (Fig 2A and 2B), consistent with the experimental observations. In line with the experimental smFRET trajectories, at the equivalent of 3 ms, 1 ms, and 0.25 ms exposure time, the MD simulations also clearly revealed two distinct FRET populations (~0.6 and 0.4 FRET efficiency) that rapidly interconverted (Fig 2A and 2B). Decreasing the time step averaging to 1 ms or 0.25 ms, revealed that the FRET efficiencies of these two states reached a maximum separation of ~0.3 FRET, corresponding to FRET efficiency values of approximately 0.65 and 0.35 FRET for open and closed conformations, respectively (Fig 2B).

Importantly, the estimated κ^2^ value in this system (0.61 ± 0.11) revealed that that the fluorophore tumbling behaviors remained within 8% of the expected value for perfect isotropic tumbling (0.66). These findings corroborate the accuracy of the experimentally derived approximations of R_0_ and κ^2^ employed for our simulations and suggest that the average positions of the LD555 and LD655 fluorophores on LIV-BP^SS^ may be modestly compacted towards their sites of attachment at the LIV-BP^SS^ surface. Such findings are consistent with the expected flexibility of the alkyl chain linker connecting the protein and fluorophore, which may be exacerbated by short-lived fluorophore-linker and/or fluorophore-protein interactions that lead to compaction of the dye center of mass towards the protein surface.

### MD simulations recapitulate population-level smFRET data

Single-molecule fluorescence and FRET experiments seek to determine how a system functions by evaluating the compositional and conformational dynamics of large ensembles of individual molecules. In the case of LIV-BP^SS^, function is inferred from the opening and closing dynamics that arise from amino acid binding and unbinding in the experimental system.

To accurately determine the mean FRET efficiencies and standard deviations of the experimentally measured FRET states, individual smFRET traces were compiled into population FRET histograms that were then fit to Gaussian distributions (Fig 3A–3C). Histograms of this kind clearly illustrate that the low- and high-FRET states of LIV-BP^SS^, which correspond to open and closed conformations, respectively, only become fully resolved at exposure times below ~5 ms in the experiments. At 5 ms exposure time high- (~0.6) and low- (~0.4) FRET states exhibit significant overlap due to the existence of exchange processes between open and closed conformations that occur on the integration time scale. Decreasing the integration time in the experiments leads to gradual resolution of two distinct FRET peaks, where the open and closed conformations exhibited ~0.65 and ~0.35 mean FRET efficiency values, respectively (Table 1).

**Table 1:** FRET efficiencies determined from Gaussian distribution of FRET distributions. Gaussian distribution used to fit histograms is defined by Eq 7, listed are the means ± variance of the low and high FRET states corresponding to experimental smFRET and MD simulation.

	Low FRET State		High FRET State	
	Experimental	Simulation	Experimental	Simulation
5 ms	0.41 ± 0.10	0.35 ± 0.06	0.61 ± 0.10	0.56 ± 0.15
3 ms	0.40 ± 0.10	0.35 ± 0.06	0.64 ± 0.07	0.56 ± 0.15
1 ms	0.36 ± 0.09	0.32 ± 0.09	0.65 ± 0.07	0.63 ± 0.11
0.5 ms	0.34 ± 0.09	0.32 ± 0.10	0.65 ± 0.07	0.63 ± 0.13

In the MD simulations, we observe a similar behavior to the experiments. In particular, the open and closed FRET efficiencies become more clearly differentiated as the estimated exposure time is decreased (Fig 3B). To identify the mean of these populations we fit the histograms of the simulated FRET data to a sum of two Gaussian distributions (Fig 3D). These fits revealed that the mean FRET efficiencies obtained from MD simulations are approximately 0.33 and 0.62 for open and closed conformations, respectively (Table 1). These computationally estimated FRET values, while slightly lower than expected from experiment—the observed differences of ~0.02 to 0.03 correspond to distances of ~1–2 Å, respectively—remain in close agreement with those estimated by smFRET (Table 1).

The modest differences in the mean FRET efficiency values between the MD and smFRET experiments may arise from a number of potential sources. This may include inaccuracies in the fluorophore-linker and fluorophore-protein contacts used to calibrate structure-based simulations, corrections used to estimate experimentally derived FRET efficiencies, or the absolute values of specific fluorophore parameters (namely R_0_) used to convert the distances observed in MD simulations to FRET efficiency.

In addition to the open and closed conformations of LIV-BP^SS^, we also observed an intermediate population in our simulations at relatively slow time regimes (3 and 5 ms) (Fig 3D). This may be akin to the blurring of low and high-FRET states observed experimentally in slow exposure regimes, (Fig 3C and 3D), where artifactual intermediates appear to arise due to the time averaging of the simulated FRET signal.

The slight difference in FRET efficiency values or time-averaging intermediates observed in MD may arise from subtle distinctions in the simulated fluorophore-linker interactions compared to those present in the fully solvated experimental setting. As the simulations performed treat hydrogen bonding and electrostatic interactions as implicit contacts, it is likely the fluorophore dynamics differ slightly from those that actually occur *in vitro*. Nonetheless, the simulated dynamics of LIV-BP^SS^ closely recapitulate those observed experimentally by smFRET. Further iterations of the simulated contacts can be explored to determine how the FRET efficiency values for open (*apo*) and closed (Leu-bound) LIV-BP^SS^ conformations vary with linker and dye composition. Such efforts are expected to inform on potential optimizations and the influence of the site of protein attachment on experimentally derived and simulated FRET efficiency values.

### Single-molecule and molecular dynamics simulated-FRET changes reflect interdomain movements in LIV-BP^SS^

We next sought to quantitatively examine the extent to which the fluctuations in FRET efficiency observed in our simulations correspond to conformational changes of the LIV-BP^SS^ protein. Due to the flexible nature of the fluorophore linkers, LD555 and LD655 can, in principle, approach each other without the protein undergoing a complete conformational transition from *apo* (open) to Leu-bound (closed) state, and *vice versa*. In other words, the fluorophores could fail to precisely track the changes in distance between the two LIV-BP^SS^ domains to which they are attached.

To assess the potential contributions of such divergences to our analyses, we sought to examine the correlation between the inter-fluorophore distance–and therefore FRET efficiencies–with the actual LIV-BP^SS^ domain movements in the simulations. While these two values are often assumed to be closely correlated, it is not obvious that they are actually correlated in light of, for example, relatively long linker lengths required for smFRET experiments. The assumption of close correlation between the fluorophore and the domain to which it is attached actually entails two implicit assumptions: (i) the residue position on the protein accurately reflects movement of an entire domain, as opposed to a local rearrangement, and (ii) the flexible linkers can be approximated by rigid bodies, fully correlated with movement of the protein.

To examine the extent to which this assumption holds for the entire domain to which the fluorophores are attached, we sought out an alternative reaction coordinate that captures the maximum amplitude of LIV-BP^SS^ conformational change, independent of any possible restrictions on labeling positions imposed by the practicalities of experiments. We chose a maximum amplitude reaction coordinate that optimizes separation of conformations on approximate free energy landscapes, aiding in estimations of barrier height. To find this reaction coordinate we measured the distance between all C_α_ atoms in domain 1 to all C_α_ atoms in domain 2. These distances were compared between the open and closed conformations to generate changes in distance, Δ, for each amino acid pair between domains 1 and 2 (S6 Fig). This analysis revealed areas of the protein that undergo the largest interdomain distance changes during conformational change.

**Fig 4:**
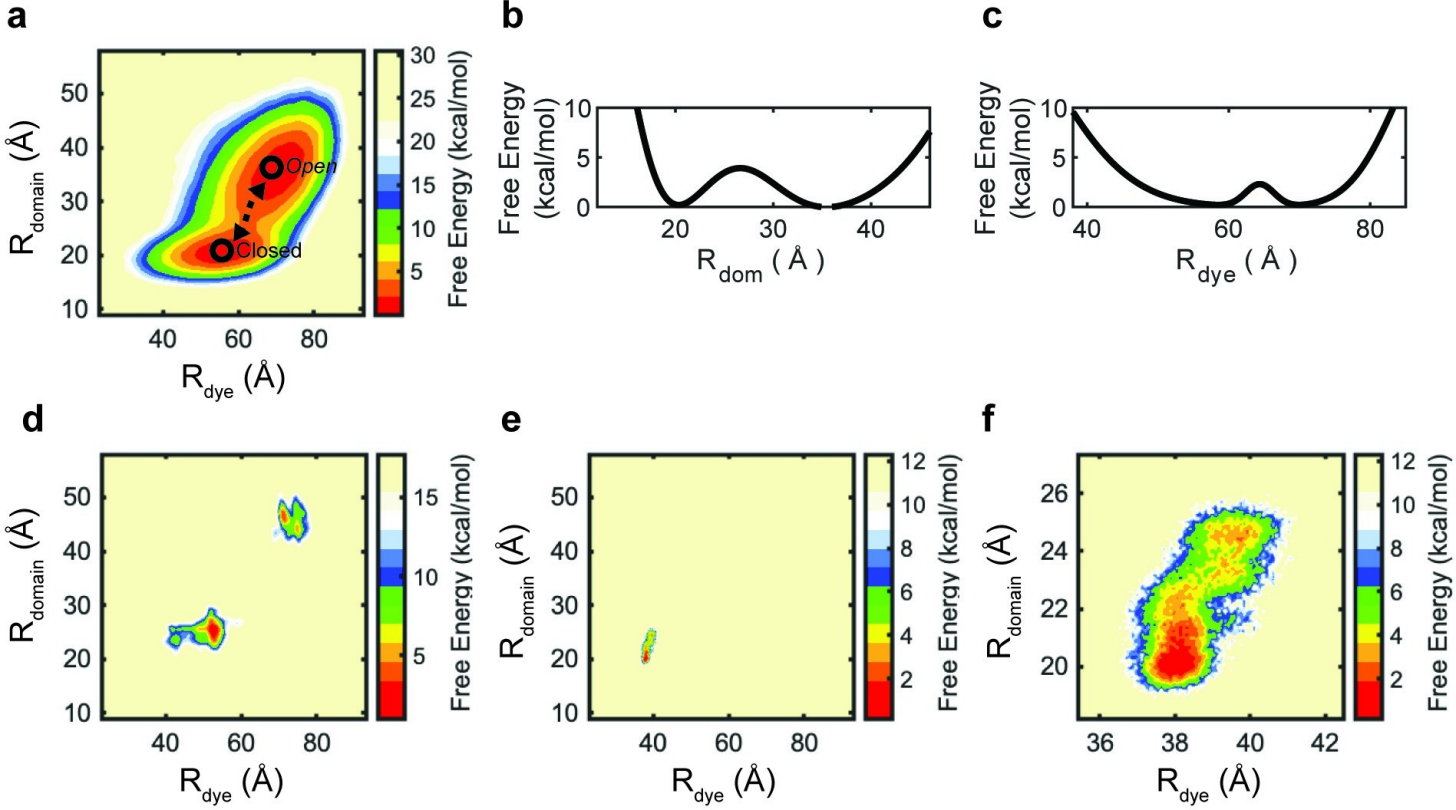
Correlation of the fluorophore and inter-domain distances of LIV-BP^SS^. (a) Boltzmann-weighted free energy landscape with reaction coordinates of inter-domain distance (R_domain_; left axis) and inter-dye distance (R_dye_; bottom axis) from 100 x 500 million-time steps structure-based simulations (50 billion-time steps). The vertical axis (scale bar at right) represents the fraction of simulation time, calculated as a relative free energy (see Methods). The centers of the energy basins refer to the *apo* and Leu-bound structures, highlighted with black circles. The Pearson correlation coefficient for the two reaction coordinates is 0.73 indicating a correlation between the estimated distances between the LIV-BP^SS^ domains and conjugated fluorophores. The barrier between the Leu-bound and *apo* states (black circles) is ~ 2–4 kcal/mol. (b) Free energy along the reaction coordinate R_domain_. (c) Free energy along the reaction coordinate R_dye._ (d-e) R_domain_ with respect to R_dye_ for explicit solvent simulations of LIV-BP^SS^ in the (d) *apo* and (e) Leu-bound states. Free energy landscapes of explicit solvent simulations are from three, 1 μs simulations, as such the separate peaks of the *apo* states are from different trajectories. (f) A zoom in on the Leu-bound free-energy landscape (panel e).

We found that the amino acid pair Ser 12 and Ala 237, displayed the largest distance change (Δ ~13 Å) between open and closed conformations. Thus, we define the alternative reaction coordinate, R_domain_, to be the distance between C_α_ atoms of Ser 12 and Ala 237. We note that, while the change in R_domain_ is larger than the change for the label positions used in the experiments, the R_domain_ states are too close (~35 ± 4 Å and ~22 ± 3 Å for open and closed states, respectively) for FRET-based experimental measurements due to practical considerations (S2 Fig). A Boltzmann-weighted approximate free energy landscape was calculated, indicating that R_domain_ and R_dye_ are correlated (Pearson’s correlation of 0.73) (Fig 4A). Here, the open conformation exhibits a mean R_dye_ value of ~69 Å and a R_domain_ of ~35 Å, whereas, the closed conformation exhibits a mean R_dye_ value of ~56 Å and a R_domain_ of ~20 Å. The 34 Å or 36 Å distance difference between R_dye_ and R_domain_ is a result of fluorophore linker length and positions 12 and 237, used to measure R_domain_, being closer together than positions 67 and 181, the sites of fluorophore conjugation in our smFRET experiments. From R_domain_ or R_dye_, the apparent barrier heights of LIV-BP^SS^ conformational change can be approximated to 3.8 kcal/mol or 2.5 kcal/mol, respectively (Fig 4B and 4C). To connect the barrier heights to rates we used the relationship between protein diffusion coefficients, rates, and free energy barriers [75,76]. As the reaction coordinate R_domain_ leads to the larger barrier height we used it to determine the rate of LIV-BP^SS^ conformational change (S2 Text). From these calculations we observed Leu-bound and *apo* LIV-BP^SS^ to undergo conformational changes at rates of 750 s^-1^ to 6000 s^-1^ and 14773 s^-1^ to 19530 s^-1^, respectively, where the lower limit is approaching the rate determined by smFRET of 210 s^-1^ (S7 Fig; S2 Table) [68]. As diffusion is determined by the degree of roughness in the landscape, the overestimation of the rate of conformational transitions by LIV-BP^SS^ is likely a result of the low degree of roughness in the free energy landscapes from structure-based model approaches. The rate difference between the *apo* and Leu-bound states is a result of the up to 10-fold difference in prefactor (S2 Table). The prefactor represents the attempt frequency, and is measured from the time dependent variance of R_domain_ in explicit solvent simulations. As the *apo* state is more dynamic, the time dependent variance of R_domain_ is larger, leading to the larger prefactor (see below) (Fig 4D–4F). The rate is proportional to the prefactor and to the exponential term (dependent on the barrier height), resulting in both making important contributions to rate estimates. Barrier height calculations are often limited by sampling; however, the all-atom structure-based potential significantly enhances sampling. On the other hand, the prefactor, determined by explicit solvent simulations, is somewhat limited in terms of sampling. Thus, longer simulations or implementation of explicit solvent enhanced sampling techniques to estimate diffusion may improve the accuracy of our methodology.

To examine the effect of finite linker length, we considered a third reaction coordinate, R_Cα_, which measures the distances between the C_α_ atoms at the labeling positions used in the smFRET experiments (positions 67 and 181). This pair exhibited a distance change (Δ) between open (~57 Å) and closed (~49 Å) conformations of approximately 7.7 Å (S6 Fig). Comparing this reaction coordinate with R_dye,_ the distance between the fluorophores (each of which is attached to a linker), we obtained a Pearson correlation coefficient of 0.8, indicating the fluorophore fluctuations correlate with domain motions of LIV-BP^SS^ (S8 Fig).

### The open state of LIV-BP^SS^ is inherently dynamic

We performed explicit solvent simulations of two systems: (i) LIV-BP^SS^ in the open state without ligand (apo) and (ii) LIV-BP^SS^ in the closed state with ligand (Leu-bound). Comparison of R_domain_ and R_dye_ for explicit solvent simulations of the *apo* state indicates that this LIV-BP^SS^ state is significantly more flexible than the Leu-bound state (Fig 4D). Three 1 μs simulations of LIV-BP^SS^ in the *apo* (open) or Leu-bound (closed) states were combined to create approximate free energy landscapes comparable to those from structure-based simulations (Fig 4D–4F). In the Leu-bound state simulation, a single conformational basin was observed with a minimum at a R_domain_ and R_dye_ of 20 Å and 38 Å, respectively (Fig 4E). Upon closer inspection, this minimum was found to exhibit some heterogeneity (Fig 4F), indicative of modest interdomain and interdye distance variations in the Leu-bound state, albeit orders of magnitude less than variations in the all-atom structure-based MD simulations.

By contrast, the *apo* state explicit solvent simulations exhibited two well-separated FRET populations, corresponding to a R_domain_ value of either ~45 Å or ~25 Å and R_dye_ values of ~75 Å and ~50 Å, respectively. As the explicit solvent simulations were initiated from the open conformation, the differences in the occupied positions for each simulation on the free energy landscape are a result of the fluctuations sampled. Close inspection of these simulations revealed that the two LIV-BP^SS^ domains can indeed approach each other in the absence of ligand to achieve a conformation similar in nature to the closed conformation (S9 Fig). Here, we note that while the two domains of LIV-BP^SS^ approach each other, they do not reach the same conformational space adopted by Leu-bound LIV-BP^SS^ (Fig 4D–4F). The trajectories of the *apo* state simulations that approach the closed conformation all initiate their transition fairly rapidly in the simulation (within the first 10 ns). Interconversion between the closed and open conformation is not observed in the explicit solvent simulations in the 1 μs sampling regime. However, the *apo* state simulations are observed to be more dynamic as reflected by width of the free energy landscape populations (Fig 4D and 4E) and the RMSD and RMSF values, which were both on average ~1.5 Å larger than the Leu-bound simulations of LIV-BP^SS^ (S10 and S11 Figs). Altogether, the results indicate that the open state is more dynamic and correspondingly more likely to interconvert between the closed and open conformations, with longer sampling.

## Discussion

Efforts to quantify the relationships between data obtained through smFRET imaging and MD simulation have the potential to provide atomic descriptions of rate-limiting, large-scale conformational changes in biological systems critical to cellular function and regulation. Atomistic-resolution information on such large-scale events are currently limited by the computational costs of explicit solvent simulations over long time scales.

To overcome these limitations, we have used a computationally efficient all-atom structure-based approach to simulate the model protein LIV-BP^SS^ that recapitulates smFRET data on the same time scale as *in vitro* experiments. The LIV-BP^SS^ system undergoes a relatively simple clamshell-like transition in converting between open and closed conformations. The integration of smFRET and simulation is not, however, limited to small proteins. In fact, it is also suited to larger and more complex systems that undergo a diversity of conformational changes potentially dominated by more than one mode. More complex systems, such as the ribosome, can be observed to undergo large-scale conformational transitions by smFRET, but the nature of these conformational changes is difficult to understand even when informed by high-resolution structural information. Our long-term goal is to employ the integration of smFRET with MD simulations to enable atomistic, structural dynamic descriptions of the complex rotations, counter rotations, swivel- and tilt-like domain movements that have been evidenced to underpin ribosome functions during translation.

As demonstrated, MD simulations of the kind described herein provide direct insight into the relationship between dynamics of a system and the observed FRET values evidenced experimentally. In doing so they have the potential to provide dynamic structural descriptions of the underlying physical barriers separating states of a system. Such efforts hold the promise of providing complete descriptions of the reaction coordinate evidenced by smFRET investigations and critical information about the nature of the conformational changes observed. At the same time, MD simulations can be used to inform on strategies to maximize smFRET signals or to investigate alternative reaction coordinates. Such strategies could involve optimization of FRET pairs utilized, given the impact on κ^2^ from the labelling position environment measured in simulations. Improvements from this symbiotic relationship between experiment and simulations can be extended to gain a deeper understanding of the fluorophore-linker, fluorophore-protein and fluorophore tumbling behaviors at their chosen sites of attachment. Ultimately, this relationship aims to delineate the most accurate FRET distance relationships possible. For instance, simulations could be employed to inform chemical synthesis efforts to alter the fluorophore linker lengths for a given FRET pair to optimize κ^2^, all of which can be performed *in silico* using the approach described above. In so doing, the functional reaction coordinate of interest can be fully revealed to the experimentalist and more informative experiments can be implemented.

Future endeavors in this area of inquiry will benefit from further advances in the experimental strategies used to site-specifically incorporate extrinsic fluorophores within compositionally diverse biomolecules. The bridge between experiment and simulation will be further enhanced by continuing imaging platform and fluorophore developments that further extend smFRET studies into the microsecond time domain. Such time scales will reduce computational burdens and ultimately afford explicit solvent simulations of smFRET data on commensurate time scales. Likewise, as computational resources extend the simulated time scales, the gap between experiment and simulation approaches will decrease. Synergistic endeavors of this kind provide a deeper understanding of complex biological systems and the structural dynamic events that occur during conformational transitions between distinct states evidenced in smFRET experiments. Continued focus on this frontier is ultimately expected to inform the developments that will enable MD simulations to recapitulate more fully biological systems of interest and extend their predictive capacity for increasingly diverse areas of inquiry.

## Methods

### Modelling of Liv-BP Conjugated to self-healing Cy3 and Cy5 fluorophores

The coordinates for the *E*. *coli* LIV-BP protein were accessed from the protein data bank, PDB ID: 1Z15 –super open form, 1Z16 –LIV-BP•Leu, 1Z17 –LIV-BP•Ile, and 1Z18 –LIV-BP•Val [71]. Positions Cys53 and Cys78 were altered to Ser to recapitulate the Liv-BP^SS^ variant rapid kinetics used in Fitzgerald *et al*. 2019 [68]. Coordinates for the self-healing fluorophores, LD555 and LD655, were designed and geometries optimized with Avogadro 1.2.0 and GAUSSIAN 09, respectively [77,78]. B3LYP theory and a 6-31G(d) basis was used for the GAUSSIAN geometry optimization [79,80]. Positions Asn67 and Asp181 of LIV-BP^SS^ were altered to Cys and the self-healing fluorophores LD555 and LD655 were conjugated to these positions, respectively, through thiol-maleimide reaction. This was repeated for the super open (*apo*), Leu, Ile, and Val bound LIV-BP complexes.

### Explicit solvent equilibrium simulations

Each of the LIV-BP^SS^ systems were solvated in a 12.0 Å SPCE water box and neutralized to a salt concentration of 100 mM KCl and 5 mM MgCl_2_ with the tleap package in AMBER18. The parameters for the self-healing LD555 and LD655 fluorophores were created in the antechamber AMBER package [82,83]. The potential energy of the water and total LIV-BP^SS^ system was minimized for 1,000 and 10,000 steps, respectively, using a steepest descent approach in AMBER18 using AMBER ff19SB parameters [81]. Each system was heated to 300 K in steps of 10 K over 50 ps using a Langevin thermostat and equilibrated for 300 ps prior to simulation. Simulations were performed with AMBER18 using Lennard-Jones interactions with a 10 Å cut-off, with periodic boundaries and the particle-mesh Ewald method [81]. The SHAKE algorithm was used for all bonds involving a constrained hydrogen. A step size of 2 fs was used in the simulations and all explicit solvent simulations were performed for a minimum of 1 μs, totaling 12 μs of aggregate simulation time.

### All atom Gō-like structure-based simulations

All atom Gō-like structure-based simulations were performed as previously described [48,66]. AMBER minimized structures of the *apo* LIV-BP^SS^ protein or bound to Leu, Ile, or Val (excluding hydrogens) were used as starting structures for structure-based models. Coordinate and topology files for structure-based models were constructed using Smog-2.2 [84]. A multi-basin Gaussian contact potential (C_ij_) described previously by Noel *et al*. 2012 [85], was used, defined by:

$$C_{ij}\left(r_{ij},r_{\alpha }^{ij},r_{\beta }^{ij}\right)=\left(1+{\left(\frac{\sigma_{NC}}{r_{ij}}\right)}^{12}\right)\left(1+G(r_{ij},r_{\alpha }^{ij})\right)\left(1+G(r_{ij},r_{\beta }^{ij})\right)-1,$$ (1)

Where, r_ij_ is the distance between atoms i and j, α and β are the open and closed conformations, respectively, σ_NC_ = 2.5 Å is the excluded volume size, and

$$G(r_{ij},r_{0}^{ij})=-Aexp\left[\frac{-{\left(r_{ij}-r_{0}^{ij}\right)}^{2}}{{\left(2\sigma \right)}^{2}}\right]$$ (2)

where, σ is the width of the gaussian well which is set to a depth of -1. With this approach both the open and closed conformations non-bonded contacts, bond-distances, and angles are set as native states. The potential (V_ij_) used for the simulations is defined by:

$$\begin{array}{c} V_{ij}=\sum_{bonds}\varepsilon_{r}{\left(r_{ij}-r_{0}\right)}^{2}+\sum_{angles}\varepsilon_{\theta }{\left(\theta_{i}-\theta_{i,0}\right)}^{2}+\sum_{impropers}\varepsilon_{xi}{\left(\chi_{i}-\chi_{i,0}\right)}^{2}\\ +\sum_{planar}\varepsilon_{xp}{\left(\chi_{i}-\chi_{i,0}\right)}^{2}+\sum_{\begin{array}{c} backbone\\ dihedrals \end{array}}\varepsilon_{BB}F_{D}(\phi_{i}-\phi_{i,0})+\sum_{\begin{array}{c} sidechain\\ dihedrals \end{array}}\varepsilon_{SC}F_{D}(\phi_{i}-\phi_{i,0})\\ +\sum_{contacts}{\varepsilon_{c}C}_{ij}(r_{ij},r_{0}^{ij})+\sum_{non-contacts}\varepsilon_{NC}{\left(\frac{\sigma_{NC}}{r}\right)}^{12} \end{array}$$ (3)

where, ε_r_ = 50 ε_0_, ε_θ_ = 40 ε_0_, ε_χi_ = 10 ε_0_, ε_χp_ = 40 ε_0_, ε_NC_ = 0.1 ε_0_, ε_0_ = 1_,_ σ_NC_ = 2.5 Å and εF_D_ = ε (1—cosΦ) + ε/2 (1 –cos 3Φ). The parameters r_0_, θ_i,0_, χ_i,0_, and Φ_i,0_ correspond to the initial bond distances, angles, planar angles, or dihedral angles, respectively, of the structure the simulation was initiated from.

All simulations were performed using a modified version of Gromacs v4.5.4, totaling 300 simulations each at 500 million time steps of size 0.002, for an aggregated 150 billion time steps [86]. The temperature of the simulations was set to 0.02–1.16 in reduced units which was maintained by Langevin dynamics to identify a temperature that reflects dynamics of the LIV-BP^SS^ protein at 300 K under explicit solvent conditions (S1 Text, S12 Fig.). A temperature of 0.3 reduced units was used for all subsequent simulations of LIV-BP^SS^. Simulations were calibrated against smFRET data to estimate the time scale of each time step ~ 0.25 ns. Additionally, the weights of the contacts were set to 0.2–0.46 to find a conformational equilibrium (S1 Text, S13 Fig.).

### Measurements of reaction coordinates

The reaction coordinate R_dye_ is the distance between the center of mass of the fluorophores, excluding linkers. To obtain a reaction coordinate describing the movements of LIV-BP^SS^ domains that displays the most variation between the open and closed conformations, the distance from the C_α_s in domain 1 was measured to each C_α_ in domain 2 of LIV-BP^SS^, culminating in a matrix of domain 1 to domain 2 Cα distances. Ser 12 and Ala 237 displayed the largest distance difference between the open and closed conformation, therefore, the distance between these amino acids was used to measure the movements of LIV-BP^SS^ domains (R_domain_).

R_dye_ was converted into a FRET efficiency value with the equation:

$$FRET=\frac{1}{1+{\left(\frac{R_{dye}}{R_{0}}\right)}^{6}}$$ (4)

where, R_0_ is the Förster distance of the fluorophore pair defined by:

$$R_{0}^{6}=\frac{2.07}{128\pi^{5}N_{A}}\frac{\kappa^{2}Q_{D}J}{n^{4}}$$ (5)

where, N_A_ is Avogadro’s constant, κ^2^ is the orientation factor, Q_D_ is the quantum yield of the donor fluorophore (0.48), and n is the refractive index of the medium and J is the spectral overlap of the two dyes. Simulations performed in implicit solvent were considered to be in a refractive index of water (1.33). The orientation factor was determined by:

$$\kappa^{2}={\left(\cos\;\theta_{AD}-3\cos\;\theta_{D}\;\cos\;\theta_{A}\right)}^{2}$$ (6)

where, θ_AD_ is the angle between the transition dipole moment vector of the acceptor and donor fluorophore, θ_D_ is the angle between the transition dipole moment vector of the donor fluorophore and the separation vector between the fluorophores, and θ_A_ is the angle between the transition dipole moment vector of the acceptor fluorophore and the separation vector between the fluorophores. The transition dipole moment vectors for the fluorophores was calculated with the Schrodinger computational suite [87].

Resulting FRET efficiencies were fit to a two gaussian distribution to identify the low and high FRET states corresponding to the *apo* and Leu-bound states. The two gaussian distribution is defined as:

$$counts=Ae^{\frac{-(x-{\bar{x}}_{a}{)}^{2}}{{2\sigma }_{a}^{2}}}+Be^{\frac{-(x-{\bar{x}}_{b}{)}^{2}}{2\sigma_{b}^{2}}}$$ (7)

where, A and B are the height of the first and second gaussian distribution, ${\bar{x}}_{a}$ and ${\bar{x}}_{b}$ are the mean of the first and second distribution, and *σ_a_* and *σ_b_* are the deviation of distribution 1 and 2.

### Analysis of structure-based simulations

Approximate Boltzmann-weighted free-energy landscapes were prepared as previously described [41]. In brief, landscapes were calculated using the g_sham package in GROMACS v4.5.4, using the equation:

$$\Delta G^{*}=-k_{B}Tln\left(\frac{P(x_{i})}{P_{max}(x)}\right)$$ (8)

where, ΔG* is the approximate free energy, P(x_i_) is the probability of being in state i, P_max_(x) is the probability of the most observed state, k_B_ is the Boltzmann constant, and T is the temperature (300 K). The free energy landscape evaluated as a function of R_dom_ and R_dye_. The barrier crossing rate (k) was calculated using the relationship between LIV-BP^SS^ kinetics and free energy profile:

$$\frac{1}{k}=\int_{R_{initial}}^{R_{final}}\;dR\;\int_{\infty }^{R}\;dR_{domain}\prime \frac{e^{\frac{\left(G(R_{domain})-G(R_{domain}\prime )\right)}{k_{B}T}}}{D(R_{domain})}$$ (9)

where, G is Gibb’s free energy, D(R_domain_) is the effective diffusion coefficient along the reaction coordinate R_domain_, and R is the reaction coordinate. The integral was simplified by using a constant value for D(R_domain_); however, the maximum and minimum diffusion coefficients were considered providing a range of estimated rates.

### Analysis of smFRET data

Experimental smFRET data were previously published [70]. In that study, smFRET imaging was performed using a custom-built total internal reflection fluorescence (TIRF) microscope using scientific complementary metal-oxide semiconductor (sCMOS) detectors [5]. LD555 was excited using a 532 nm laser (Laser Quantum) and imaged at the specified time resolution. The fluorescence from LD555 and LD655 was separated using a T635lpxr dichoric (Chroma) and projected onto two Flash 4.0 v2 sCMOS cameras (Hamamatsu) using a MultiCam LS device (Cairn). Microfluidic imaging chambers passivated with a mixture of PEG and biotin-PEG were incubated for 5 min each with 0.8 μM streptavidin (Invitrogen) and 10 nM biotin-tris-NTA-Ni^2+^ [68,70,88]. His-tagged LIV-BP^SS^ (a construct with native disulfide residues removed and labeled at engineered cysteine residues in positions 67 and 181) was surface immobilized via the His-tag:Ni^2+^ interaction in for 2 minutes. All experiments were performed with 30 mM Tris (pH 7) and 150 mM NaCl buffer and with leucine present at 4.5 μM, which corresponds to the K_D_ for LIV-BP^SS^ where bound and unbound states are equally occupied. Wide-field TIRF movies acquired in this way were analyzed with SPARTAN [5], including corrections for donor to acceptor crosstalk, gamma, and acceptor direct excitation [89,90].

### Fluorescence quantum yield and anisotropy measurements

Absolute fluorescence quantum yield of LD555 labeled LIVBP^SS^ was measured in a FluoTime 300 spectrometer using integrating sphere accessories (PicoQuant GmbH, Berlin) and a 300 W xenon excitation lamp. The absorbance of the sample at the excitation wavelength was adjusted to 0.02 in a Shimadzu UV-2600 spectrometer to minimize re-absorption of emitted photons. For the absolute quantum yield measurements, the sample was photoexcited at 517 nm and the photons were detected from 512 to 700 nm.

Steady-state fluorescence anisotropy of LD555-LIVBP^SS^ was also recorded in FluoTime 300 spectrometer using 300 W xenon lamp as an excitation source. The sample was photoexcited at 517 nm and the anisotropy was recorded in the emission range 550–650 nm. All the measurements were carried out in a buffer containing 30 mM Tris (pH 7) and 150 mM NaCl at room temperature using standard 1 cm path length quartz cuvettes (Starna Cells, Inc.). The data were analyzed in EasyTau software (PicoQuant GmbH, Berlin).

## Supporting information

S1 Fig Multibasin Gaussian Potential used to define contacts for the native state. Representation of the potential (V_ij_) for each native state, with regards to the distance between an atom contact pair, defined by Eq 3. This example of a contact pair has a minimum V_ij_ at 5 Å and at 10 Å, corresponding to the atom distances in the first and second native state. The barrier between the minima can be defined with σ_1_ and σ_2_ and the basin depth defined by A in Eq 2.
(TIF)

S2 Fig The distances of LIV-BP domains and conjugated fluorophores. The distance between the Cα of Ser12 and Ala 237 (R_domain_) and the distance between the center of mass of the fluorophores (R_dye_) as measured during a structure-based simulation (1x10^6^ time steps binned for 0.25 ms sampling). Interconversion between the Leu-bound and *apo* states can be observed in both the R_dye_ and R_domain_ reaction coordinates as R_domain_ transitions from a 29.5 (closed) to a 33 Å (open) state and R_dye_ transitions from a 56 (closed) to 69 (open) Å state during a single simulation.
(TIF)

S3 Fig Fluorophore transition dipole moments. Emission and absorption dipoles of central chromophore structures of self-healing fluorophores, respectively, at B3LYP/6-311G(d,p) level of theory. The core of the LD555 and LD655 fluorophores used in the present study is identical to Cy3 and Cy5, respectively, and thus serve as a reasonable proxy for these calculations.
(TIF)

S4 Fig Simulated smFRET in the absence of dye-linker interactions. At 3, 1, and 0.25 ms sampling in the absence of dye-dye interactions structure-based simulations overestimate the FRET efficiency for both the closed and open state.
(TIF)

S5 Fig Population level comparison of simulated FRET efficiencies. (a) Contour plots of FRET efficiencies calculated from MD simulations in the presence of dye-linker interactions. (b) Contour plots of FRET efficiencies calculated from MD simulations in the absence of dye-linker interactions.
(TIF)

S6 Fig Identification of an optimal reaction coordinate describing LIV-BP conformational change. The distance between each Cα in domain 1 was measured to each Cα in domain 2 of LIV-BP for both the *apo* and Leu-bound states. The difference in the distances between the *apo* and Leu-bound states were then plotted on the heat map as Δ distances. This matrix reveals the amino acid pair whose distance changes the most during conformational change of LIV-BP. The Cys 67 and Cys 181 amino acid pair that was used to conjugate fluorophores to LIV-BP are highlighted as the FRET Pair.
(TIF)

S7 Fig Connecting the free energy of LIV-BP domain movements with the rate of conformational change. (a) The means square displacement of the reaction coordinate R_domain_ of LIV-BP^SS^ measured from 1 μs explicit solvent simulations, correlated with lag time. (b) Free energy function (eq S2) used to connect the free energy landscape to the rate of LIV-BP^SS^ conformational change through changing barrier height. (c) The correlation of the rate and prefactor (Ca) in relation to barrier height for LIV-BP^SS^ conformational change using the lower (red) and upper (black) estimate of diffusion of 0.3 μm^2^s^-1^ and 7.8 μm^2^s^-1^.
(TIF)

S8 Fig Correlation of the fluorophore and fluorophore conjugation sites to describe inter-domain dynamics of LIV-BP. (a) Boltzmann-weighted free energy landscape with reaction coordinates of inter-domain distance (R_cα_; left axis) and inter-dye distance (R_dye_; bottom axis) from 100 x 500 million-time steps structure-based simulations (50 billion-time steps). The vertical axis (scale bar at right) represents the fraction of simulation time, calculated as a relative free energy (see Methods). The centers of the energy basins refer to the *apo* and Leu-bound structures, highlighted with black circles. The Pearson correlation coefficient for the two reaction coordinates is 0.8 indicating a correlation between the estimated distances between the LIV-BP domains and conjugated fluorophores. The barrier between the Leu-bound and *apo* states (black circles) is ~ 2–3 kcal/mol. (b-c) R_Cα_ with respect to R_dye_ for explicit solvent simulations of LIV-BP in the (b) open and (c) closed states. Free energy landscapes of explicit solvent simulations are from three, 1 μs simulations, as such the separate peaks of the open and closed configurations are from different trajectories. (d) A zoom in on the Leu-bound free-energy landscape (panel c).
(TIF)

S9 Fig Flexibility and closure of LIV-BP domains in the absence of ligand. LIV-BP in the *apo* state at 0 and 1 μs to display the spontaneous closure of domain 1 (pink) and domain 2 (blue). Fluorophores are conjugated to the protein and are represented as sticks. At 0 μs simulation there are few intramolecular contacts between the fluorophores and their linkers, at 1μs the number of intramolecular contacts has increased, compacting the fluorophores.
(TIF)

S10 Fig Root-mean-square deviation (RMSD) of LIV-BP in explicit solvent simulations. One microsecond simulations of LIV-BP in the (A) *apo* (green), (B) Leu-bound (red), (C) Ile-bound (blue), (D) Val-bound (red). Simulations were performed in triplicate. All simulations adopted an RMSD of < 6 Å.
(TIF)

S11 Fig Root-mean-square fluctuation (RMSF) of LIV-BP explicit solvent simulations. RMSF of a 1 μs simulations of LIV-BP in the *apo* (green), Leu-bound (red), Ile-bound (blue), and Val-bound (pink) states.
(TIF)

S12 Fig Temperature determination for structure-based simulations. (A) RMSF of structure-based simulations at temperatures ranging from 0.02 to 0.5 reduced units. The RMSF of explicit solvent simulations of the *apo* and Leu-bound state are highlighted as black and grey, respectively. Similar RMSF trends are observed between the explicit solvent and structure-based simulations. (B) Average RMSF of the structure-based simulations with respect to RMSF. There is a linear relationship (y = 3.987x+0.6072, R^2^ = 0.9587) between the temperature of the structure-based simulations and RMSF. The average RMSF of explicit solvent simulations of the *Apo* and Leu-bound states are highlighted. From the linear fit a temperature of 0.3 reduced units, which is exactly between the Leu and *Apo* explicit solvent simulations, was chosen to use for the structure-based simulations.
(TIF)

S13 Fig Leu-bound state specific contacts impact on conformational changes. Leu-bound state specific contacts were reweighted 0.2–0.46 to identify weight to facilitate LIV-BP conformational changes.
(TIF)

S1 Table Calculate κ^2^ values from structure based-simulations and explicit solvent simulations. κ^2^ for structure-based simulations was calculated from 100, 500-million time step simulations. κ^2^ for explicit solvent simulations was calculated from 3, 1 μs simulations. The reported error is the standard deviation between simulation replicates.
(DOCX)

S2 Table Parameters calculated from explicit solvent simulations to correlate free energy barriers and rates. (DOCX)

S1 Text Structure-based simulation parameterization. (DOCX)

S2 Text Connecting free energy landscapes and LIV-BPSS conformational change rate. (DOCX)
