# Supplementary figures and images for: Quantitative comparison between sub-millisecond time resolution single-molecule FRET measurements and 10-second molecular simulations of a biosensor protein
Source: PLoS Comput Biol. 2020 Nov 5;16(11):e1008293. doi: 10.1371/journal.pcbi.1008293 (PMC7643941; doi:10.1371/journal.pcbi.1008293)

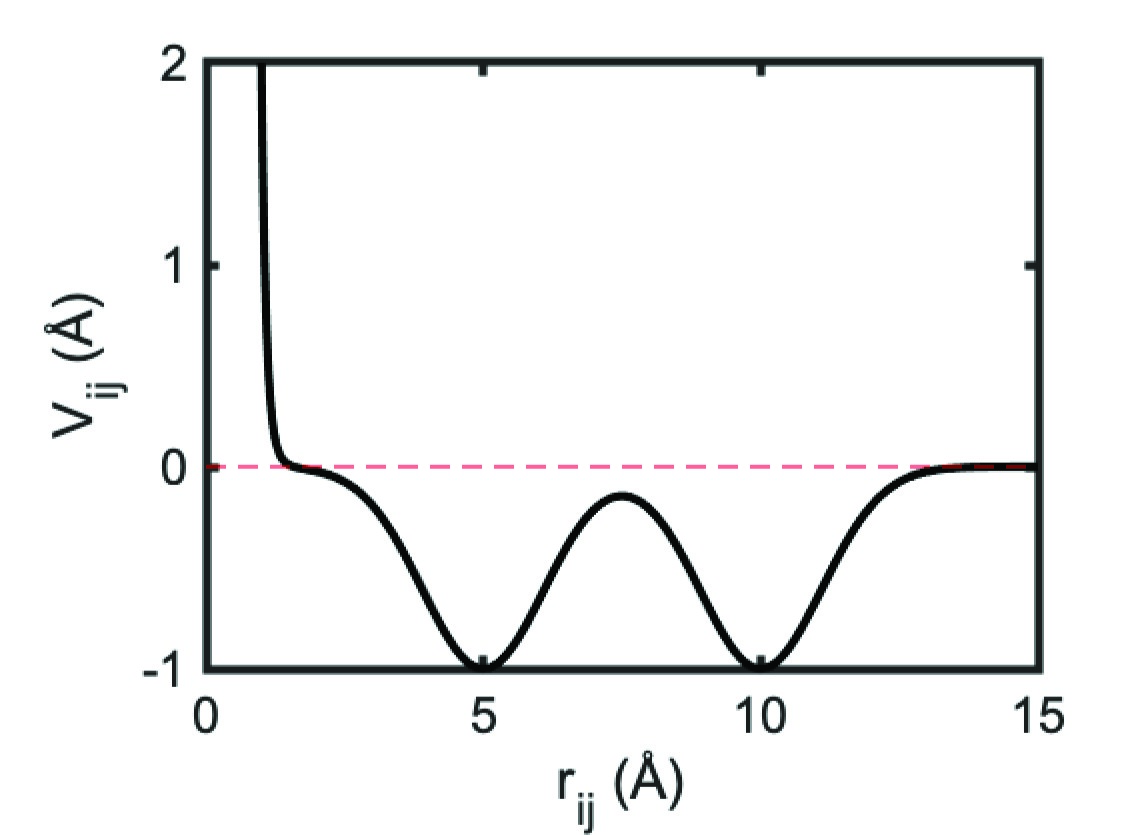

Supplement: S1 Fig — Representation of the potential (Vij) for each native state, with regards to the distance between an atom contact pair, defined by Eq 3. This example of a contact pair has a minimum Vij at 5 Å and at 10 Å, corresponding to the atom distances in the first and second native state. The barrier between the minima can be defined with σ1 and σ2 and the basin depth defined by A in Eq 2. (TIF) [file pcbi.1008293.s001.tif]

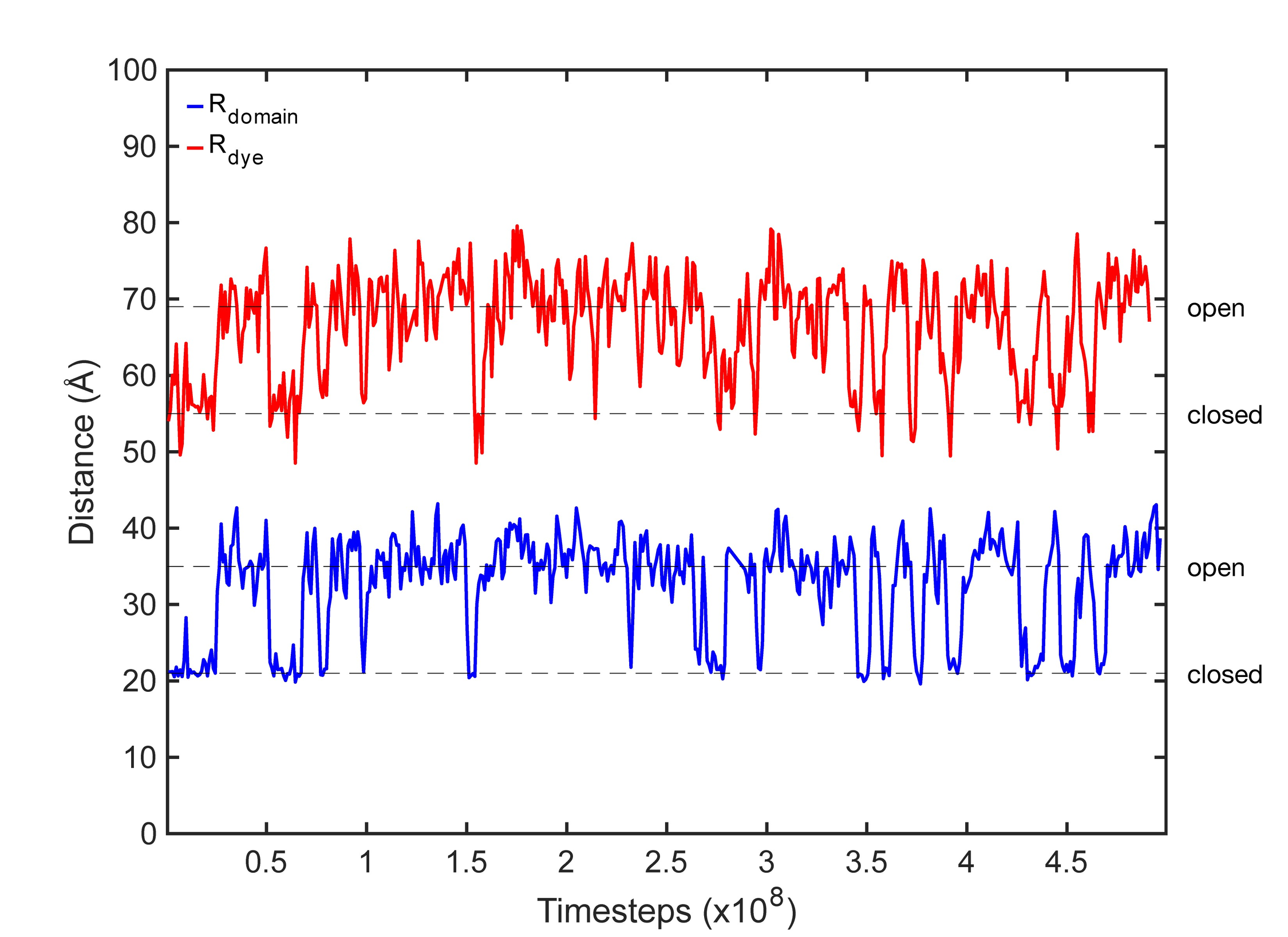

Supplement: S2 Fig — The distance between the Cα of Ser12 and Ala 237 (Rdomain) and the distance between the center of mass of the fluorophores (Rdye) as measured during a structure-based simulation (1x10^6^ time steps binned for 0.25 ms sampling). Interconversion between the Leu-bound and apo states can be observed in both the Rdye and Rdomain reaction coordinates as Rdomain transitions from a 29.5 (closed) to a 33 Å (open) state and Rdye transitions from a 56 (closed) to 69 (open) Å state during a single simulation. (TIF) [file pcbi.1008293.s002.tif]

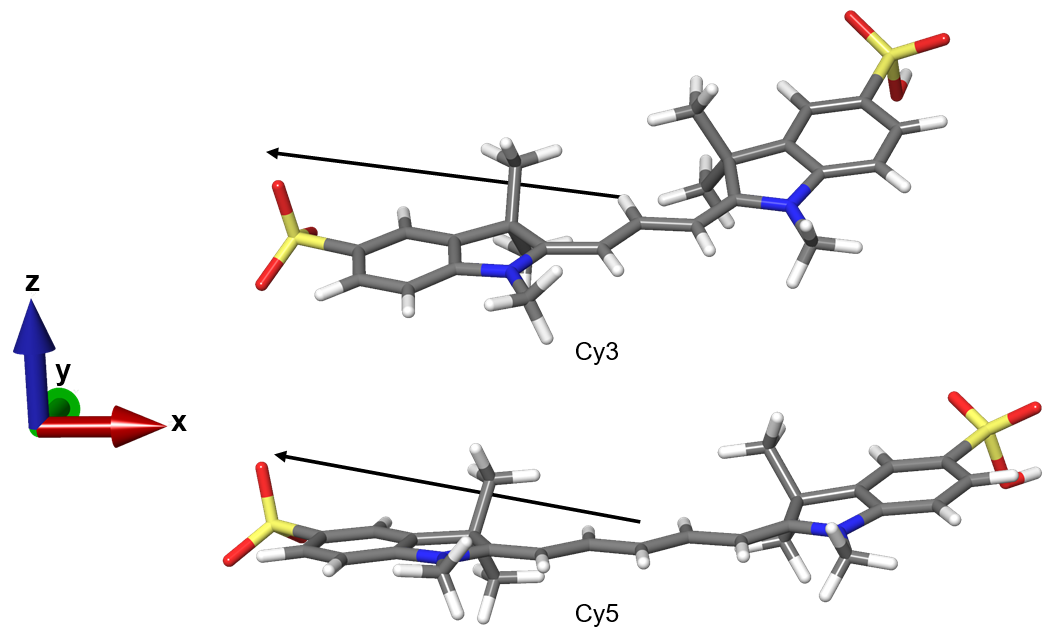

Supplement: S3 Fig — Emission and absorption dipoles of central chromophore structures of self-healing fluorophores, respectively, at B3LYP/6-311G(d,p) level of theory. The core of the LD555 and LD655 fluorophores used in the present study is identical to Cy3 and Cy5, respectively, and thus serve as a reasonable proxy for these calculations. (TIF) [file pcbi.1008293.s003.tif]

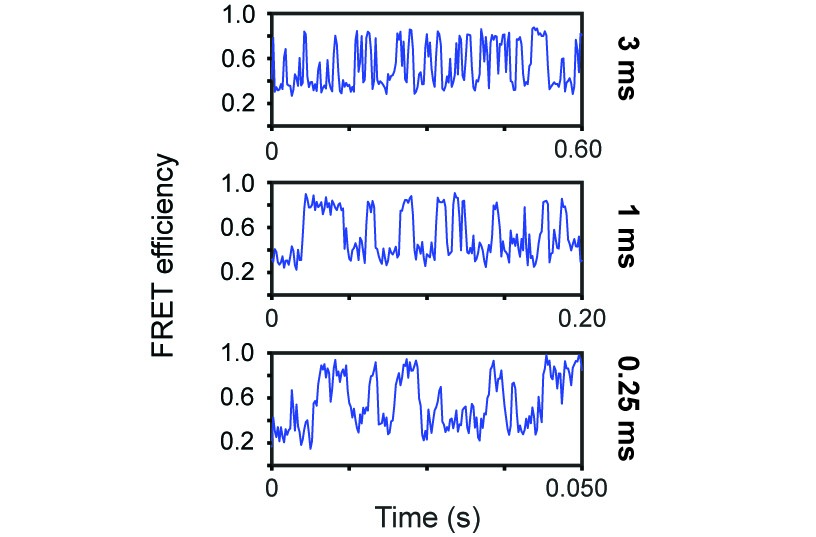

Supplement: S4 Fig — At 3, 1, and 0.25 ms sampling in the absence of dye-dye interactions structure-based simulations overestimate the FRET efficiency for both the closed and open state. (TIF) [file pcbi.1008293.s004.tif]

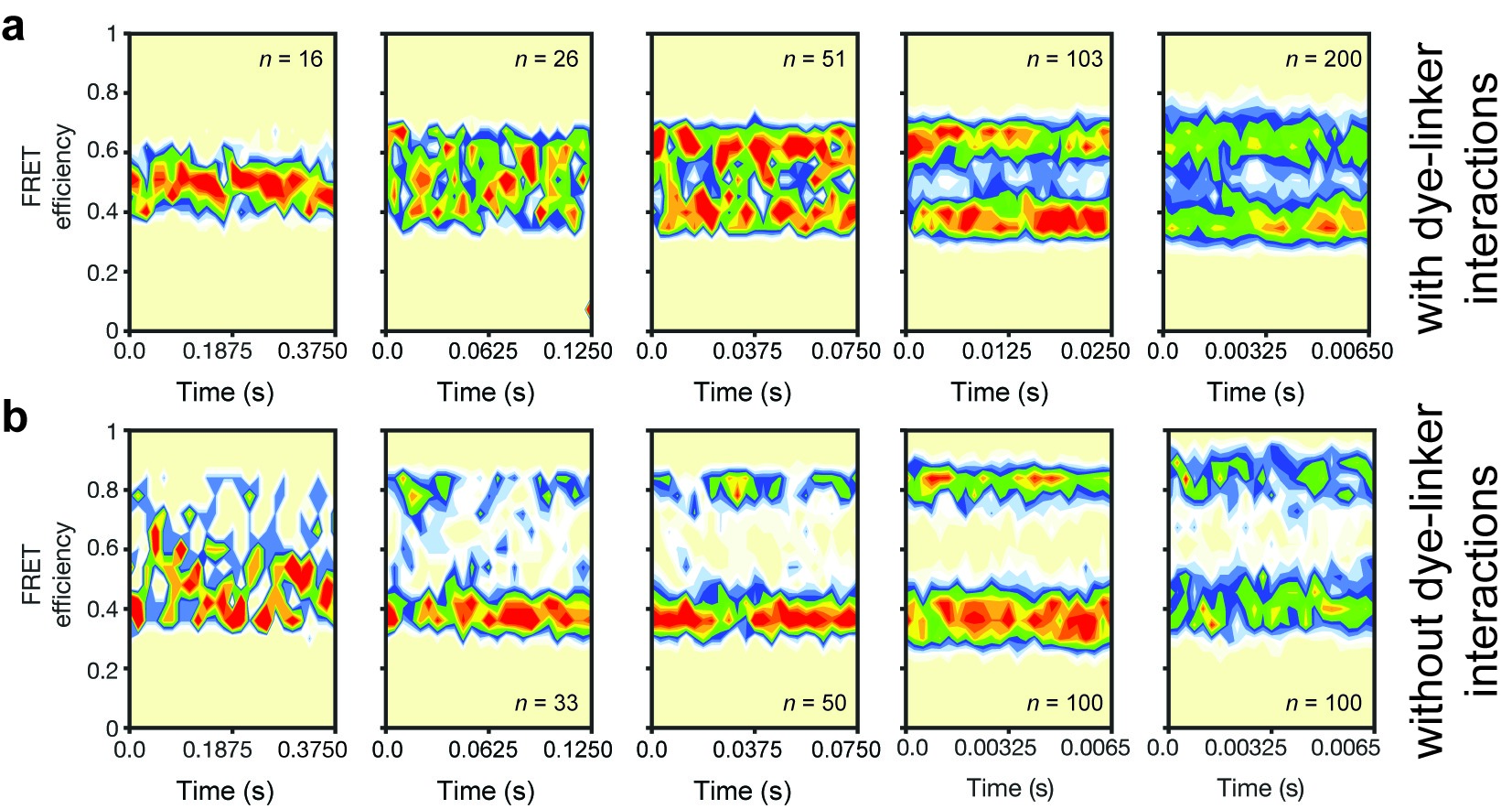

Supplement: S5 Fig — (a) Contour plots of FRET efficiencies calculated from MD simulations in the presence of dye-linker interactions. (b) Contour plots of FRET efficiencies calculated from MD simulations in the absence of dye-linker interactions. (TIF) [file pcbi.1008293.s005.tif]

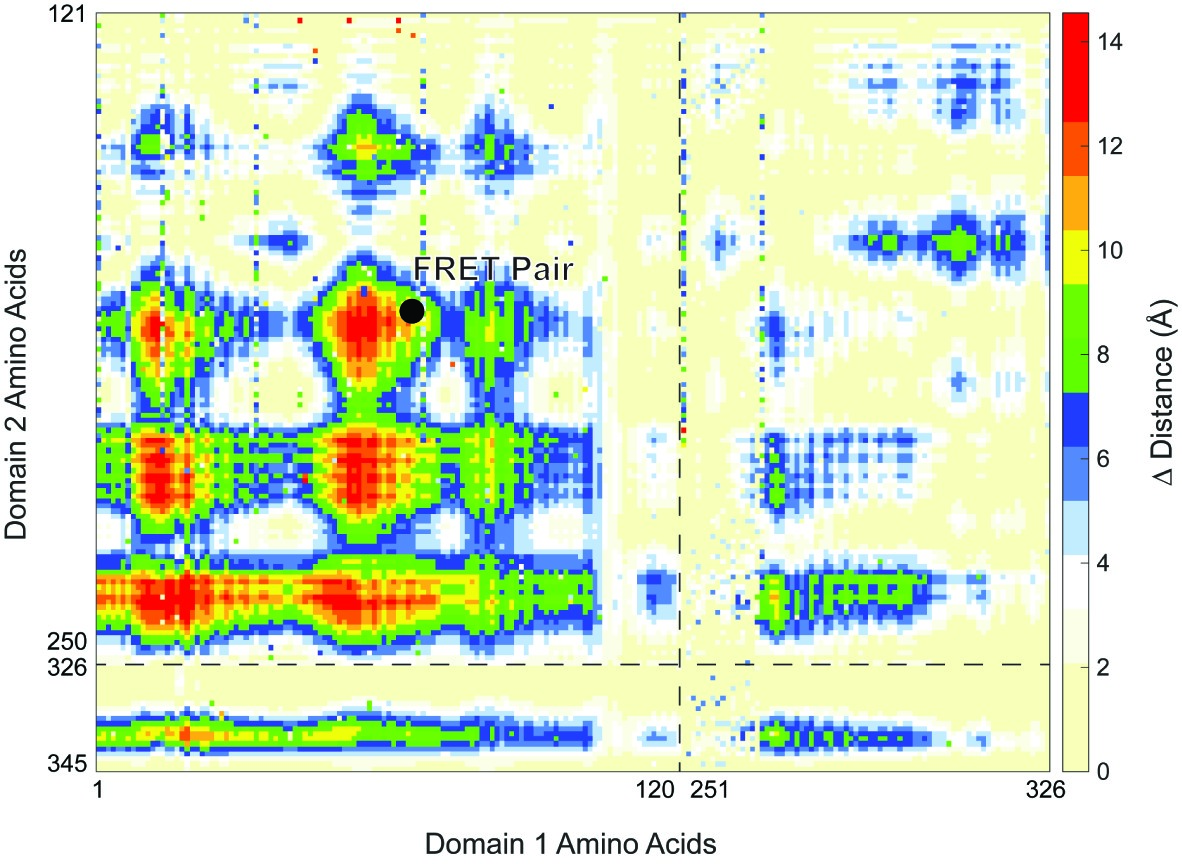

Supplement: S6 Fig — The distance between each Cα in domain 1 was measured to each Cα in domain 2 of LIV-BP for both the apo and Leu-bound states. The difference in the distances between the apo and Leu-bound states were then plotted on the heat map as Δ distances. This matrix reveals the amino acid pair whose distance changes the most during conformational change of LIV-BP. The Cys 67 and Cys 181 amino acid pair that was used to conjugate fluorophores to LIV-BP are highlighted as the FRET Pair. (TIF) [file pcbi.1008293.s006.tif]

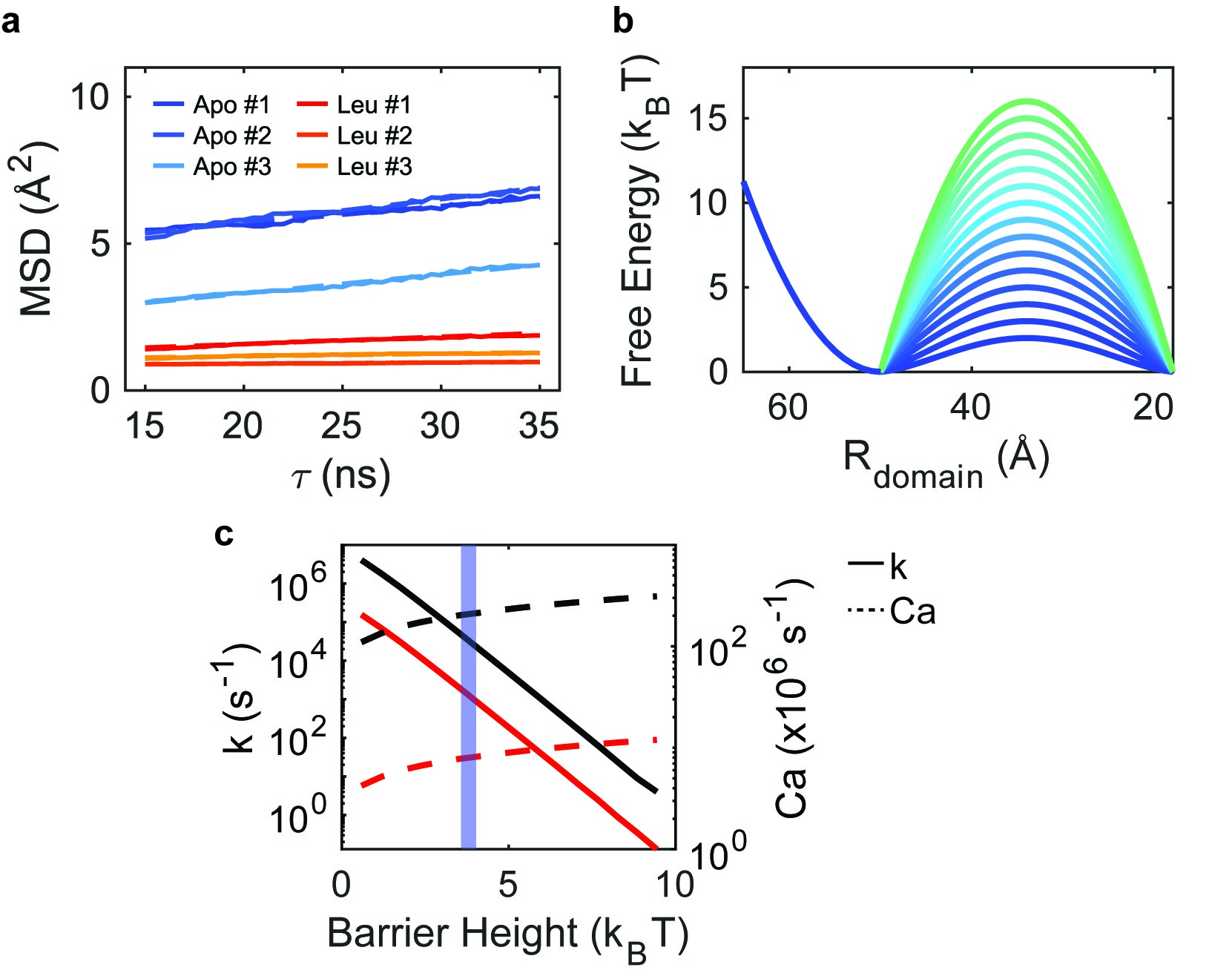

Supplement: S7 Fig — (a) The means square displacement of the reaction coordinate Rdomain of LIV-BPSS measured from 1 μs explicit solvent simulations, correlated with lag time. (b) Free energy function (eq S2) used to connect the free energy landscape to the rate of LIV-BPSS conformational change through changing barrier height. (c) The correlation of the rate and prefactor (Ca) in relation to barrier height for LIV-BPSS conformational change using the lower (red) and upper (black) estimate of diffusion of 0.3 μm2s-1 and 7.8 μm2s-1. (TIF) [file pcbi.1008293.s007.tif]

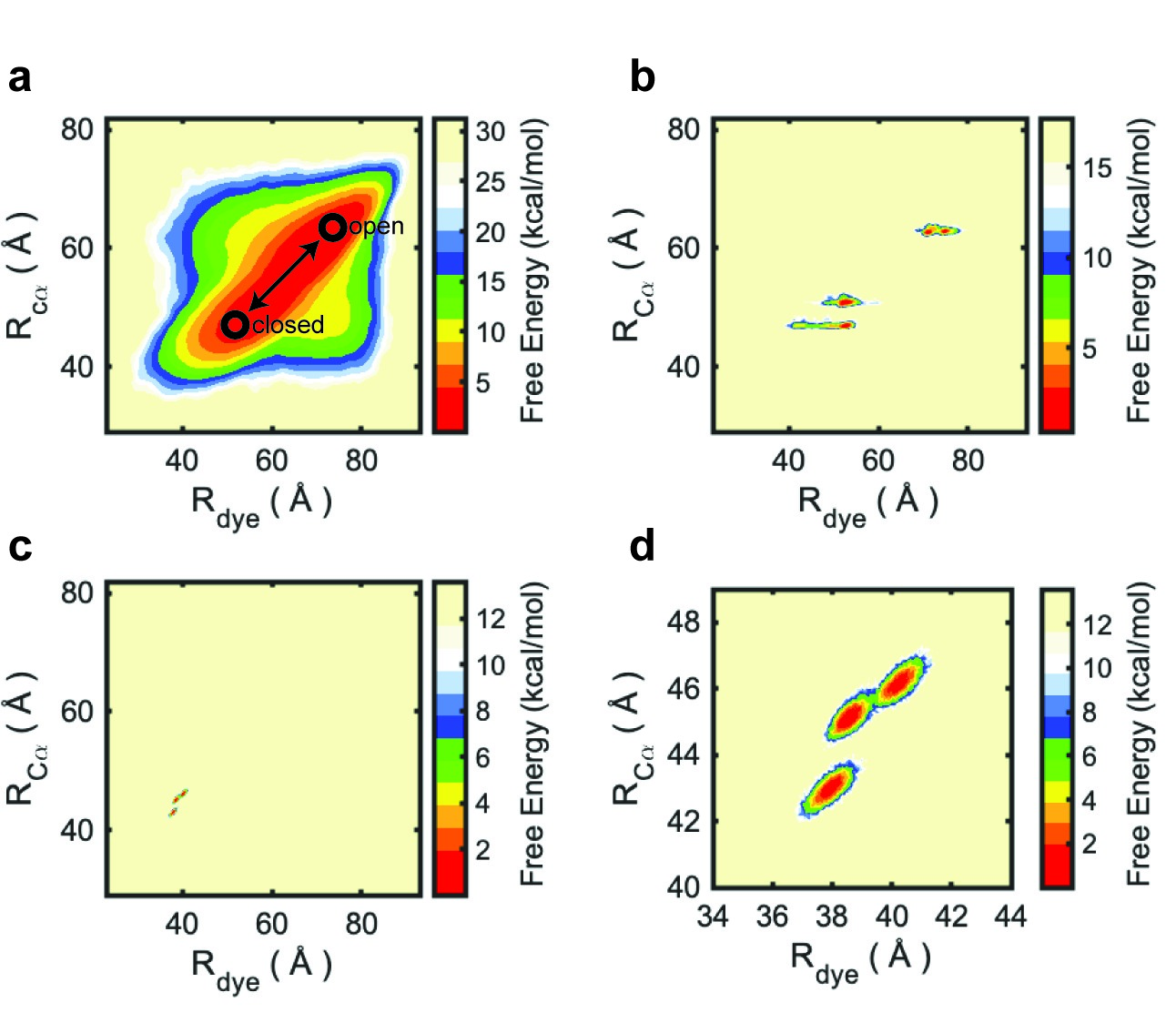

Supplement: S8 Fig — (a) Boltzmann-weighted free energy landscape with reaction coordinates of inter-domain distance (Rcα; left axis) and inter-dye distance (Rdye; bottom axis) from 100 x 500 million-time steps structure-based simulations (50 billion-time steps). The vertical axis (scale bar at right) represents the fraction of simulation time, calculated as a relative free energy (see Methods). The centers of the energy basins refer to the apo and Leu-bound structures, highlighted with black circles. The Pearson correlation coefficient for the two reaction coordinates is 0.8 indicating a correlation between the estimated distances between the LIV-BP domains and conjugated fluorophores. The barrier between the Leu-bound and apo states (black circles) is ~ 2–3 kcal/mol. (b-c) RCα with respect to Rdye for explicit solvent simulations of LIV-BP in the (b) open and (c) closed states. Free energy landscapes of explicit solvent simulations are from three, 1 μs simulations, as such the separate peaks of the open and closed configurations are from different trajectories. (d) A zoom in on the Leu-bound free-energy landscape (panel c). (TIF) [file pcbi.1008293.s008.tif]

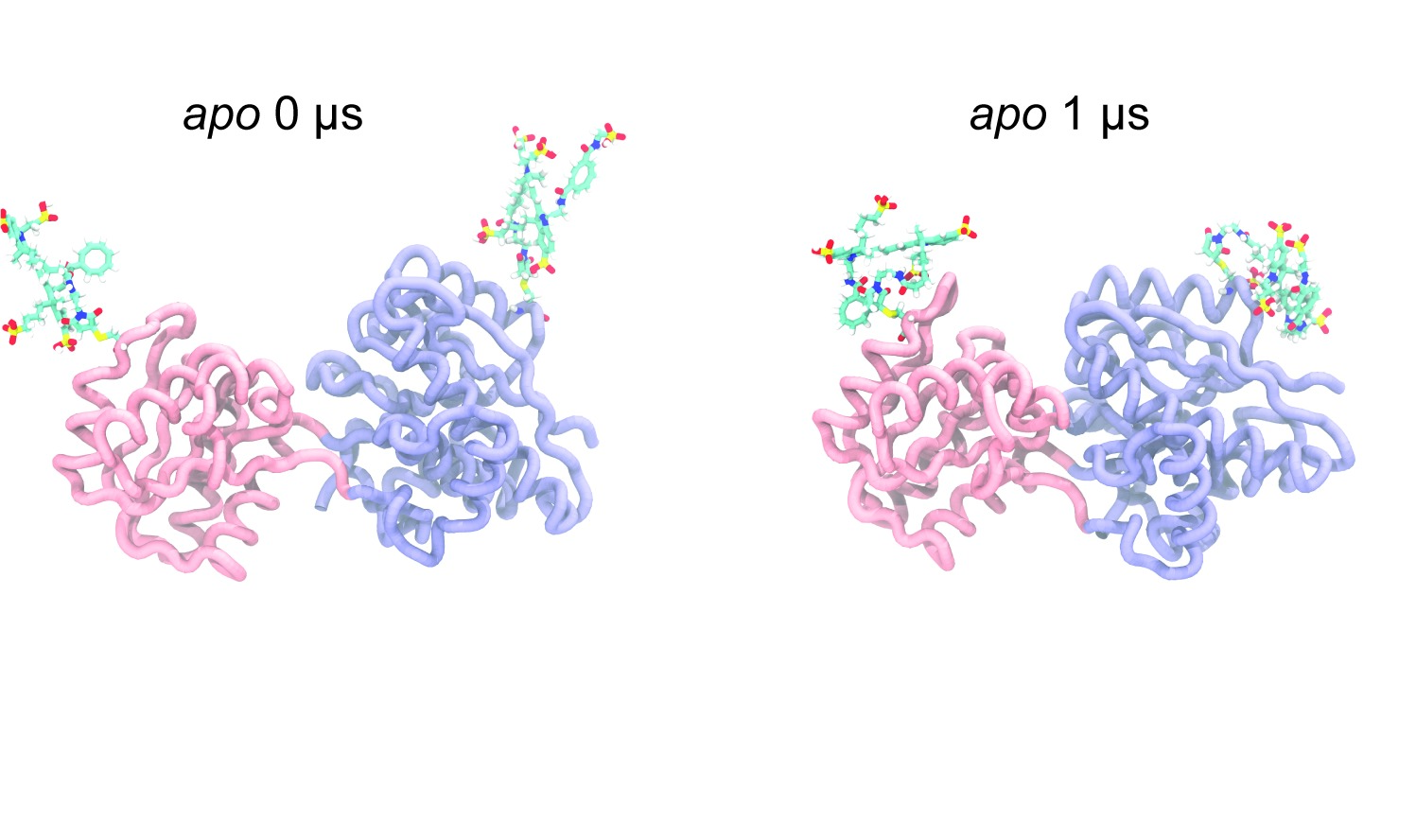

Supplement: S9 Fig — LIV-BP in the apo state at 0 and 1 μs to display the spontaneous closure of domain 1 (pink) and domain 2 (blue). Fluorophores are conjugated to the protein and are represented as sticks. At 0 μs simulation there are few intramolecular contacts between the fluorophores and their linkers, at 1μs the number of intramolecular contacts has increased, compacting the fluorophores. (TIF) [file pcbi.1008293.s009.tif]

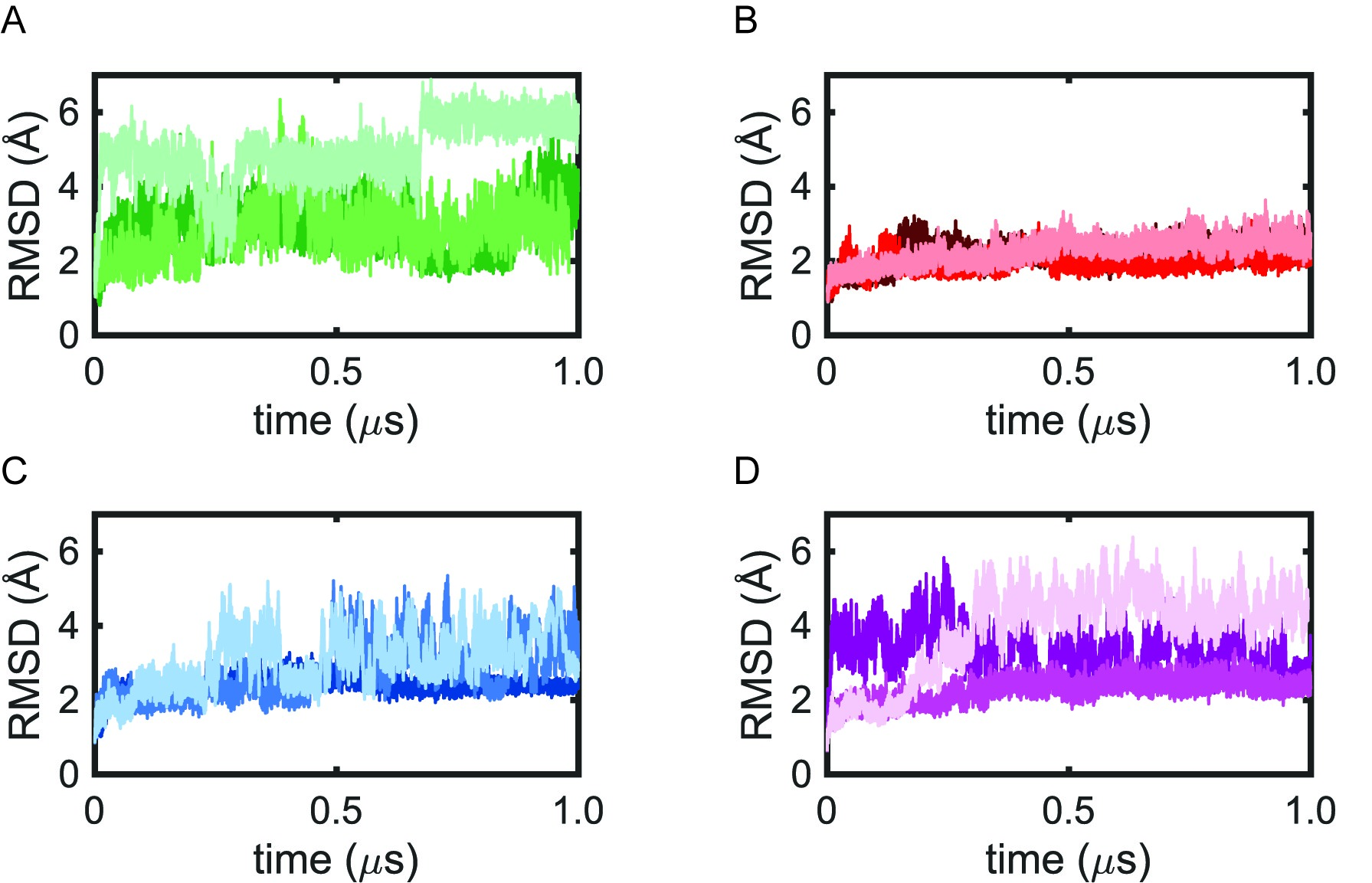

Supplement: S10 Fig — One microsecond simulations of LIV-BP in the (A) apo (green), (B) Leu-bound (red), (C) Ile-bound (blue), (D) Val-bound (red). Simulations were performed in triplicate. All simulations adopted an RMSD of < 6 Å. (TIF) [file pcbi.1008293.s010.tif]

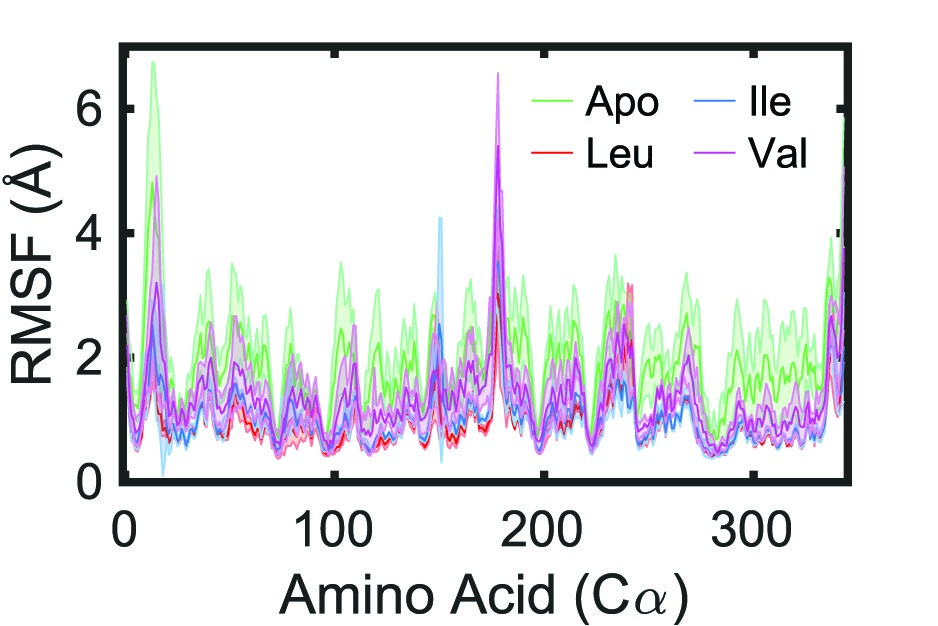

Supplement: S11 Fig — RMSF of a 1 μs simulations of LIV-BP in the apo (green), Leu-bound (red), Ile-bound (blue), and Val-bound (pink) states. (TIF) [file pcbi.1008293.s011.tif]

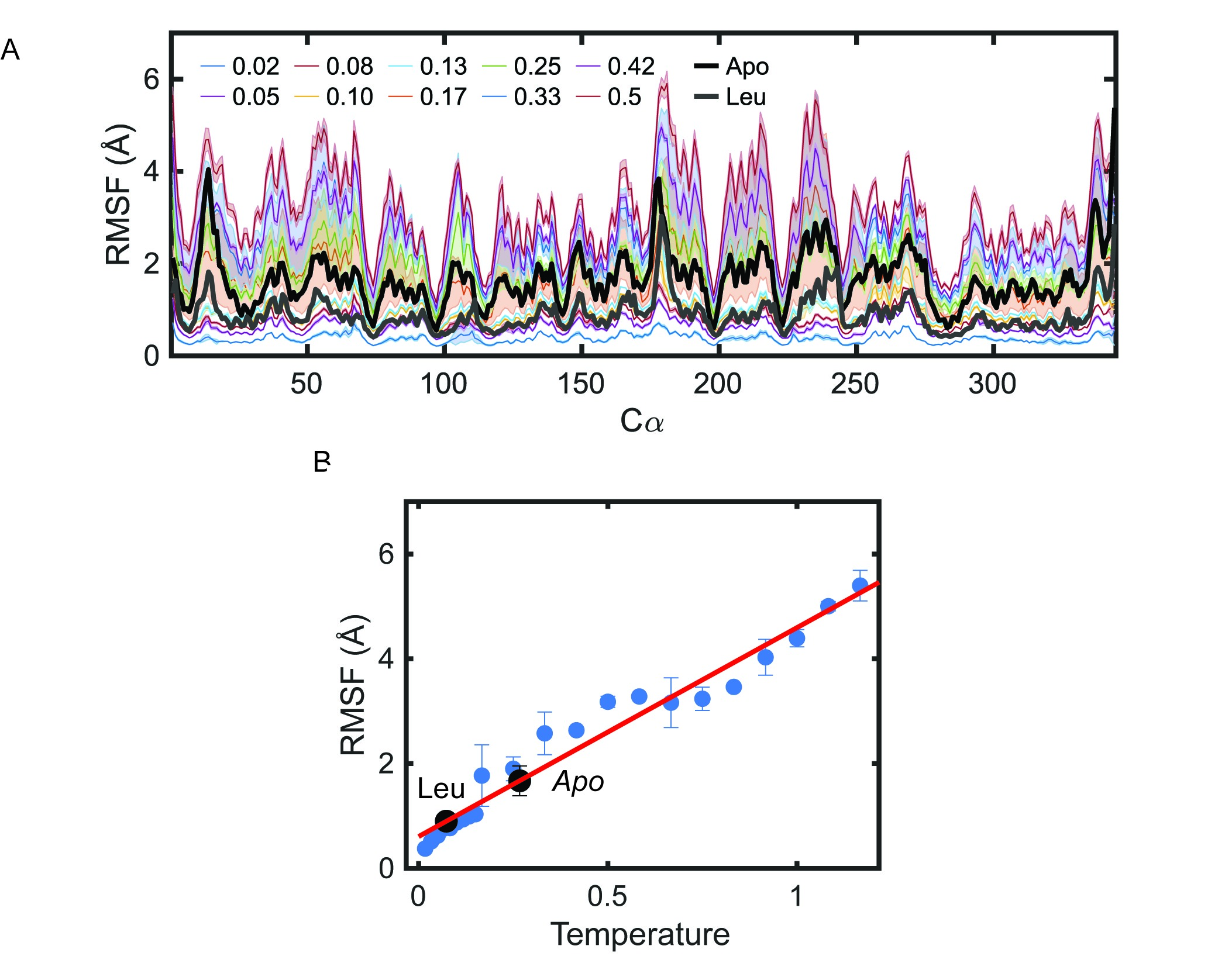

Supplement: S12 Fig — (A) RMSF of structure-based simulations at temperatures ranging from 0.02 to 0.5 reduced units. The RMSF of explicit solvent simulations of the apo and Leu-bound state are highlighted as black and grey, respectively. Similar RMSF trends are observed between the explicit solvent and structure-based simulations. (B) Average RMSF of the structure-based simulations with respect to RMSF. There is a linear relationship (y = 3.987x+0.6072, R2 = 0.9587) between the temperature of the structure-based simulations and RMSF. The average RMSF of explicit solvent simulations of the Apo and Leu-bound states are highlighted. From the linear fit a temperature of 0.3 reduced units, which is exactly between the Leu and Apo explicit solvent simulations, was chosen to use for the structure-based simulations. (TIF) [file pcbi.1008293.s012.tif]

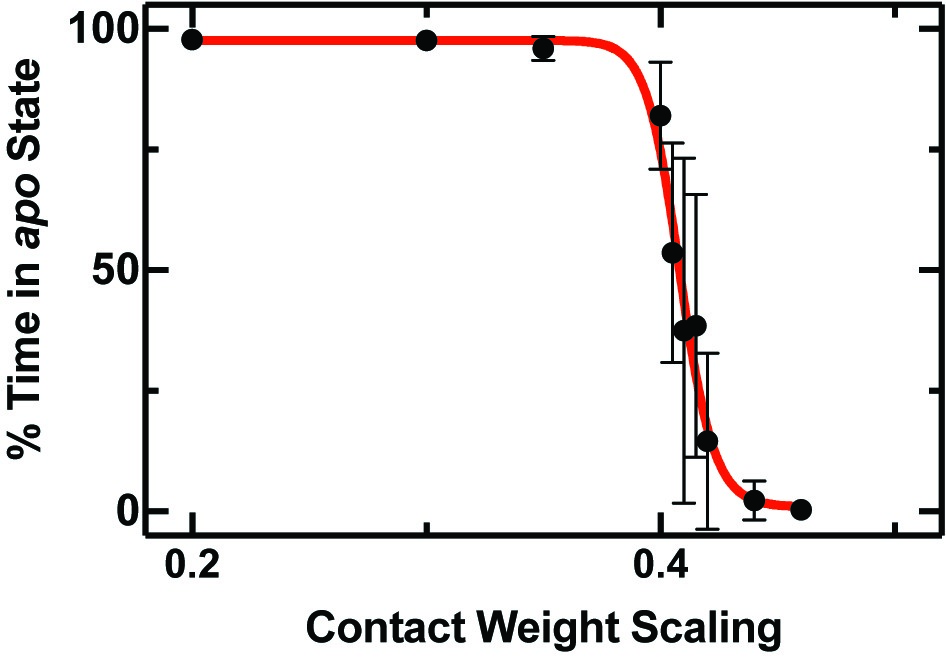

Supplement: S13 Fig — Leu-bound state specific contacts were reweighted 0.2–0.46 to identify weight to facilitate LIV-BP conformational changes. (TIF) [file pcbi.1008293.s013.tif]
