# Supplementary material for: Quantitative comparison between sub-millisecond time resolution single-molecule FRET measurements and 10-second molecular simulations of a biosensor protein
Source: PLoS Comput Biol. 2020 Nov 5;16(11):e1008293. doi: 10.1371/journal.pcbi.1008293 (PMC7643941; doi:10.1371/journal.pcbi.1008293)
Supplement: S1 Table — κ2 for structure-based simulations was calculated from 100, 500-million time step simulations. κ2 for explicit solvent simulations was calculated from 3, 1 μs simulations. The reported error is the standard deviation between simulation replicates. (DOCX) [file pcbi.1008293.s014.docx]

**Table S1. Calculate κ^2^ values from structure based-simulations and explicit solvent simulations.** κ^2^ for structure-based simulations was calculated from 100, 500-million time step simulations. κ^2^ for explicit solvent simulations was calculated from 3, 1 μs simulations. The reported error is the standard deviation between simulation replicates.

| Simulation | κ^2^ | Standard Deviation |
| --- | --- | --- |
| Structure-based no dye-linker interactions | 0.58 | 0.22 |
| Structure-based with dye-linker interactions | 0.61 | 0.11 |
| Explicit solvent open conformation | 0.45 | 0.19 |
| Explicit solvent closed conformation | 0.41 | 0.10 |
