# Supplementary material for: Quantitative comparison between sub-millisecond time resolution single-molecule FRET measurements and 10-second molecular simulations of a biosensor protein
Source: PLoS Comput Biol. 2020 Nov 5;16(11):e1008293. doi: 10.1371/journal.pcbi.1008293 (PMC7643941; doi:10.1371/journal.pcbi.1008293)
Supplement: S2 Table — (DOCX) [file pcbi.1008293.s015.docx]

**Table S2. Parameters calculated from explicit solvent simulations to correlate free energy barriers and rates.**

|  | <\|ΔR_t_-R_0_\|^2^> (intercept) (Å^2^) | <\|ΔR_t_-R_0_\|^2^> (slope) (Å^2^ns^-1^) | <\|ΔR_t_-R_0_\|^2^>  (R^2^) | Diffusion (μm^2^s^-1)^ | Rate at 3.8 kcal/mol (s^-1^) | Prefactor at 3.8 kcal/mol (C_a_) (10^6^ s^-1^) |
| --- | --- | --- | --- | --- | --- | --- |
| *Apo* #1 | 4.23 | 0.059 | 0.96 | 5.9 | 14773 | 16.2 |
| *Apo* #2 | 4.18 | 0.078 | 0.97 | 7.8 | 19530 | 21.4 |
| *Apo* #3 | 2.01 | 0.065 | 0.98 | 6.5 | 16275 | 17.8 |
| Leu #1 | 1.09 | 0.024 | 0.97 | 2.4 | 6000 | 6.6 |
| Leu #2 | 0.83 | 0.003 | 0.96 | 0.3 | 751 | 0.8 |
| Leu #3 | 0.99 | 0.009 | 0.9 | 0.9 | 2253 | 2.5 |
