# Supplementary material for: Quantitative comparison between sub-millisecond time resolution single-molecule FRET measurements and 10-second molecular simulations of a biosensor protein
Source: PLoS Comput Biol. 2020 Nov 5;16(11):e1008293. doi: 10.1371/journal.pcbi.1008293 (PMC7643941; doi:10.1371/journal.pcbi.1008293)
Supplement: S1 Text — (DOCX) [file pcbi.1008293.s016.docx]

**Supporting information Text 1**

**Supporting Results**

**Structure-based simulation parameterization**

Structure-based simulations utilizing a double Gaussian distribution for contact potentials require parameters to be optimized before accurate simulations can be performed. First the temperature, defined in reduced units, was optimized. Here, we varied the temperature of the structure-based simulations from 0.02 to 1.16 in reduced units to find a temperature that reports a Cα RMSF in the all-atom structure-based simulations similar to that observed in explicit-solvent simulations (S11a-b Figs). Next, the strength of the contacts was considered. A 4 Å cutoff was used to define a contact pair and the strength of a contact was determined by the distance between the atom pair. Initial simulations in the absence of contact scaling revealed that the Leu-bound state was over stabilized and there were no observed transitions between the Leu-bound and *apo* state. Therefore, to reduce the stability of the Leu-bound state and to observe conformational changes between the two basins, the contacts of the Leu-bound state were varied to find an optimal scaling of the specific contacts in the closed state basin (S12 Fig). At a scaling of 0.405, conformational changes between the *apo* and Leu-bound state could be readily evidenced (Figs 2b, and S2).

A feature of this type of simulation is the near equivalent contribution each native contact has to the stability of a basin. The Leu in the ligand binding pocket only contributes a few contacts to the Leu-bound state, in comparison to the native contacts found throughout the rest of the protein. Therefore, using this approach transitions some transitions to the ligand bound stand were observed in the absence of Leu binding. In this regard we refer to the conformations of LIV-BP as the ‘open’ and ‘closed’ conformations.
