# Supplementary material for: Quantitative comparison between sub-millisecond time resolution single-molecule FRET measurements and 10-second molecular simulations of a biosensor protein
Source: PLoS Comput Biol. 2020 Nov 5;16(11):e1008293. doi: 10.1371/journal.pcbi.1008293 (PMC7643941; doi:10.1371/journal.pcbi.1008293)
Supplement: S2 Text — (DOCX) [file pcbi.1008293.s017.docx]

**Supporting Information Text 2**

**Connecting free energy landscapes and LIV-BP^SS^ conformational change rate**

Rates of LIV-BP^SS^ conformational change were correlated to the free energy barrier height (Fig. 4a) using the relationship in eq 9 and from R_domain_ in explicit solvent simulations. As the barrier height of R_domain_ (3.8 kcal/mol) was higher than that of R_dye_ (2.5 kcal/mol), we used R_domain_ as the reaction coordinate to calculate the rate of LIV-BP^SS^ conformational change. The diffusion coefficient (D) of R_domain_ was determined as previously described, with the equation:

$D= \lim_{t\to t_{D}} \left( \frac{\delta}{\delta t} \right)\frac{\left\langle\left| \Delta R\left( t+\tau\right)-R\left( t \right) \right|^{2} \right\rangle}{2}$, S1

where <|ΔR_t_-R_0_|^2^> is the mean squared displacement of the reaction coordinate, τ is the time separation between frames, and t_D_ is the timescale where R_domain_ is considered diffusive within explicit solvent simulations (i.e., when there is a linear relationship between <|ΔR_t_-R_0_|^2^> and τ)[1]. The slope of the linear fit for the mean square displacement in relation to τ (S13a Fig.) indicates the diffusion coefficient (S3 Table).

The integral in equation 9 was solved using a functional form for free energy of:

$\Delta G\left( R \right)=\left\{ \begin{matrix} \Delta G_{TSE}\left[ \frac{-2\left( R_{domain}-34 \right)^{2}}{{10}^{2}}+\frac{\left( R_{domain}-34 \right)^{4}}{{10}^{4}} \right] \\ \frac{\left( R-34 \right)^{2}}{20} \end{matrix} \right.\begin{matrix} if R<50 Å \\ if R>50 Å \end{matrix}$ S2

Where, ΔG_TSE_ is the barrier height of the transition state (S13b Fig.). Previous reports have indicated that alternative functional forms have little effect on the resulting correlations between rate and free energy [2]. Using a range of diffusion coefficients from 0.3 to 7.8 μm^2^s^-1^ the rate of LIV-BP^SS^ conformational change can be estimated to be between 750 s^-1^ and 19280 s^-1^ (S13c Fig.; S3 Table).

The diffusion coefficient was also used to determine the relationship between the prefactor, or the frequency of attempts to undergo conformational change (C_a_), and the free energy landscape with equation 3:

$C_{a}=\frac{e^{\beta\Delta G_{TSE}}}{\int_{R_{initial}}^{R_{final}} dR\int_{\infty}^{R} dR_{domain}'\frac{e^{\left( \beta G\left( R_{domain} \right)-\beta G({R_{domain}}^{'}) \right)}}{D(R_{domain})}}$. S3

This relationship indicates that LIV-BP^SS^ attempts to undergo conformational change at a frequency of 1-21μs^-1^ (S13c Fig.; S3 Table).

**References**

1. Whitford PC, Blanchard SC, Cate JHD, Sanbonmatsu KY. Connecting the kinetics and energy landscape of tRNA translocation on the ribosome. PLoS Comput Biol. 2013;9(3):e1003003.

2. Whitford PC, Onuchic JN, Sanbonmatsu KY. Connecting energy landscapes with experimental rates for aminoacyl-tRNA accommodation in the ribosome. J Am Chem Soc. 2010;132:13170-1.
